# Polyphenolic Profile and Antioxidant and Aortic Endothelium Effect of *Michay* (*Berberis congestiflora* Gay) Collected in the Araucanía Region of Chile

**DOI:** 10.3390/plants15030352

**Published:** 2026-01-23

**Authors:** Fredi Cifuentes, Javier Palacios, Astrid Lavado, Javier Romero-Parra, Adrián Paredes, Mario J. Simirgiotis

**Affiliations:** 1 Laboratorio de Fisiología Experimental, Instituto Antofagasta, Universidad de Antofagasta, Antofagasta 1270300, Chile; fredi.cifuentes@uantof.cl; 2 Departamento Biomédico, Facultad Ciencias de la Salud, Universidad de Antofagasta, Antofagasta 1240000, Chile; astrid.lavado@uantof.cl; 3 Laboratorio de Bioquímica Aplicada, Química y Farmacia, Facultad de Ciencias de la Salud, Universidad Arturo Prat, Iquique 1110939, Chile; 4 Departamento de Química Orgánica y Fisicoquímica, Facultad de Ciencias Químicas y Farmacéuticas, Universidad de Chile, Santiago 8380494, Chile; javier.romero@ciq.uchile.cl; 5 Laboratorio de Química Biológica, Instituto Antofagasta, Universidad de Antofagasta, Antofagasta 1270300, Chile; adrian.paredes@uantof.cl; 6 Departamento de Química, Facultad de Ciencias Básicas, Universidad de Antofagasta, Antofagasta 1240000, Chile; 7 Instituto de Farmacia, Facultad de Ciencias, Universidad Austral de Chile, Valdivia 5090000, Chile

**Keywords:** endemic Chilean berries, *Berberis*, Berberidaceae, antioxidant capacity, docking calculations, enzyme inhibition, cardiovascular properties

## Abstract

Berries are an excellent source of bioactive compounds, particularly polyphenols, and have been widely used in folk medicine by the Mapuche people of southern Chile. In this study, a hydroalcoholic extract of *Berberis congestiflora* Gay (BE) was analyzed to determine its phytochemical composition and to evaluate its antioxidant capacity, vasorelaxant effects in rat aortas, and inhibitory activity on enzymes related to chronic non-communicable diseases, including exploration of a possible vasodilatory mechanism in isolated rat aortas. Antioxidant activity was assessed using Oxygen Radical Absorbance Capacity (ORAC), DPPH (2,2-diphenyl-1-picrylhydrazyl), and ABTS (2,2-azinobis-(3-ethylbenzothiazolin-6-sulfonic acid)) radical scavenging assays, as well as ferric reducing antioxidant power (FRAP). Vascular responses to the Berberis extract were studied using isometric tension recordings in an ex vivo rat thoracic aortic ring model, and the chemical constituents of BE were identified for the first time by HPLC-DAD-MS. The extract itself produced a dose-dependent contraction at 100 and 1000 µg/mL and induced relaxation in phenylephrine-precontracted aortas at the same concentrations, with a maximum contraction of 71% and maximum relaxation of 70% at 1000 µg/mL. Mechanistically, the extract triggered calcium-mediated contraction primarily through calcium release from the sarcoplasmic reticulum and, to a lesser degree, via extracellular Ca^2+^ influx, while its relaxant effect depended on an intact endothelium and activation of the NO/cGMP pathway. In addition, the extract showed inhibitory activity against cholinesterase, glucosidase, and amylase, with IC_50_ values of 7.33 ± 0.32, 243.23 ± 0.3, and 27.21 ± 0.03 µg/mL, respectively, and docking studies were carried out for selected berry compounds. Overall, these findings indicate that these berries are a rich source of bioactive constituents with antioxidant properties and endothelium-dependent vasodilator effects, supporting their traditional use and highlighting their potential as enzyme inhibitors and as promising candidates for the development of phytotherapeutic products, particularly as supplements for chronic disease management.

## 1. Introduction

**Figure 1:**
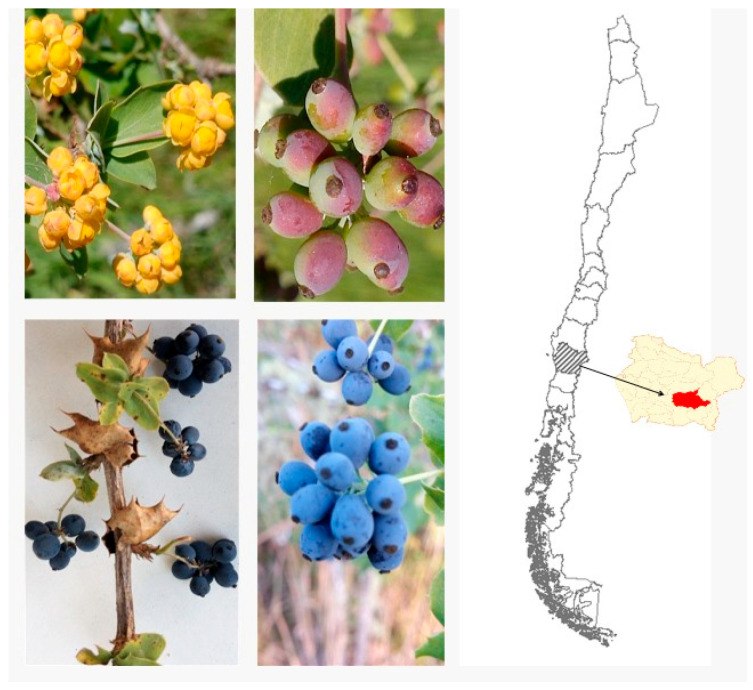
Immature *Berberis congestiflora Gay*, (**upper**), mature fruits (**lower**), and place of collection in Chile (map zone highlighted in red).

The Chilean terroir hosts a diversity of unique species, including several that produce edible berries rich in potentially beneficial compounds. However, these native species are increasingly threatened by the impacts of climate change, expanding human populations, and the pressures of the timber industry [1,2]. Previous research on Chilean endemic berries has revealed the presence of diverse phenolic compounds, including anthocyanins. For example, in *Azara dentata* Ruiz & Pav., various phenolics were identified by UHPLC-DAD-TIMS-TOF-MS, and the extract showed inhibitory activity against specific enzymes associated with chronic non-communicable diseases (CNCDs) [3]. In several other dark berries, numerous anthocyanin glycosides were detected and quantified for the first time [4], while phenolic compounds from Mapuche canelo fruits were also characterized for the first time and exhibited confirmed anti-enzymatic properties related to CNCDs [5]. In traditional Mapuche medicine, species of the genus Berberis have long been used for their hypoglycemic, hepatoprotective, anti-inflammatory, and anti-carcinogenic properties, effects that are partly attributed to their berberine content [6,7,8,9,10,11]. *Berberis congestiflora* Gay, locally known as michei or Michay (Figure 1), is a shrub that can reach up to 3 m in height, bearing leathery, glabrous leaves with entire or spiny margins, five–seven pairs of clustered spines, and an oval to elliptical blade with revolute edges. Its yellow flowers are arranged in racemes of about 25 flowers, each 2.5–3 mm long, and give rise to globose, dark blue berries of approximately 6 mm in diameter, each containing four–five black seeds. Although *B. congestiflora* itself has not been extensively studied, evidence from other Berberis species indicates a rich and diverse phytochemical profile in leaves and berries, including benzylisoquinoline and aporphine alkaloids (such as berberine, palmatine, jatrorrhizine, columbamine, and O-methylisofalicberine), hydroxycinnamic acids (e.g., caffeic and ferulic acids), anthocyanins (such as delphinidin and cyanidin glycosides), and flavonoids (including quercetin and kaempferol glycosides such as quercetin-3-O-glucoside and rutin), all associated with diverse biological activities [6,7,8]. *Berberis congestiflora* occurs from the Santiago Metropolitan Region to the Los Ríos Region in Chile, and phenolic acids, alkaloids, and flavonols have already been identified in its leaves [12]. Recent work has underscored the pharmacological potential of Chilean Berberis species: Reyes-Farias et al. (2015) showed that extracts from *Aristotelia chilensis* (maqui) and *Berberis microphylla* (calafate) exert marked inhibitory effects on inflammatory responses [13], while Martínez et al. (1997) isolated from *Berberis chilensis* the alkaloid O-methylisofalicberine (O-MI), whose structure is closely related to alkaloids previously described as Ca^2+^-channel antagonists [14]. Nevertheless, the edible fruits of Chilean Berberis remain only sparsely explored in terms of their chemistry and bioactivity, leaving considerable potential for the discovery of new medicinal compounds and for refining chemotaxonomic relationships within the genus.

## 2. Results and Discussion

The mature fruits of *Berberis congestiflora* were collected during February 2017 in the Region of Araucanía, Chile. Hydroalcoholic extracts were prepared and analyzed regarding their components as well as several bioactivities commented on below.

### 2.1. Antioxidant Activity and Content of Phenolics and Flavonoids

Oxidative stress generates reactive oxygen species (ROS)—for example, hydroxyl radicals—and nonradical oxidants like hydrogen peroxide [15]. These agents readily attack cellular constituents—amino acids, sugars, lipids, proteins, and nucleic acids—oxidizing them and thereby initiating disease processes or degrading the quality of foods and cosmetics [16]. A range of analytical assays have been developed to quantify the antioxidant capacity of natural products and gauge their suitability as protective agents. In this study, the total phenolic compound content (TPC), total anthocyanin content (TAC), and total flavonoid content (TFC) were measured using spectrometry (76.35 mg GAE, 32.26 mg c3g, and 63.2 QE per g dry weight, respectively), with TPC being higher than that of Valdivian *A. dentata* berries (57 mg GAE/g dry weight) [3], while the total anthocyanins were deemed to be high, as measured using spectroscopy, compared to the Chilean blueberries (21.42 mg/g dry fruits) [17]. The Oxygen Radical Absorbance Capacity (ORAC) assay uses fluorescence to deliver a highly sensitive measure of antioxidant activity [18], whereas the ABTS method offers excellent reproducibility by quantifying an extract’s ability to donate hydrogen atoms and convert the ABTS radical into a stable, nonradical species [19]. In this study, the *BE* extract achieved an IC_50_ of 5.32 ± 0.5 μg/mL and 6.78 ± 0.04 μg/mL by the DPPH (2,2-diphenyl-1-picrylhydrazyl) and ABTS (2,2-azinobis-(3-ethylbenzothioazolin-6-sulfonic acid) antiradical methods, respectively. This antioxidant capacity of the extract can be compared with the antioxidant potency of natural antioxidants used commercially (gallic acid DPPH: IC_50_ = 2.32 ± 0.5 µg/mL; quercetin: IC_50_ = 12.23 ± 0.8 µg/mL). Furthermore, the ferric reducing antioxidant power (FRAP) of *Michay* fruits (148.7 ± 0.03 μmol Trolox/g dry fruit, respectively) was higher than that of berries from Chilean *Berberis microphilla* and from the blueberries *Vaccinium corimbosum* (124.4 μmol and 96.15 Trolox/g dry fruit, respectively) [17] (Table 1). The ORAC of *Michay* fruits (175.9 ± 3.43 μmol Trolox/g dry fruit) was close to that published for *Ugni molinae* berries (222 μmol Trolox/g dry fruit) [20]. Given these activities, the extract could be incorporated into dietary supplements or food products as an antioxidant additive to protect them from oxidation or formulated into skincare products to help counteract the damaging effects of free radicals.

### 2.2. B. congestiflora Extract Causes Contraction in Rat Aortas

**Figure 2:**
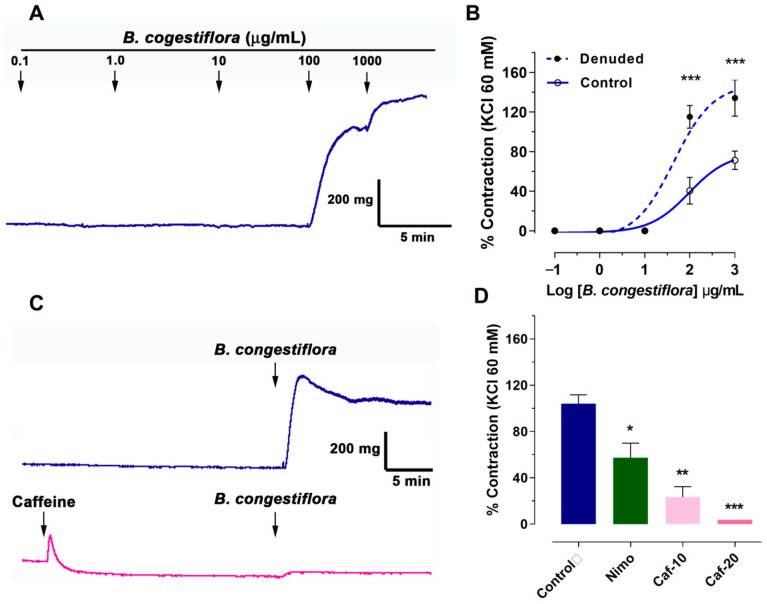
Contractile effects of *B. congestiflora* on vascular tone. (**A**) Representative trace showing the contractile response of intact aortic rings exposed to 0.1–1000 μg/mL *B. congestiflora*. (**B**) Concentration-dependent contraction of *B. congestiflora* in intact (control) and endothelium-denuded (denuded) aortic rings. Vascular contraction is expressed as a percentage of the submaximum contraction induced by 60 mM KCl. (**C**) Representative trace showing the contractile response of 1000 μg/mL *B. congestiflora* on intact aortic rings without or with 20 mM caffeine. (**D**) Effect of 20 min preincubation with 10^−4^ M nimodipine (Nimo), 10 mM caffeine (Caf-10), and 20 mM caffeine (Caf-20). Data are presented as mean ± SEM (*n* = 5). * *p* < 0.05, ** *p* < 0.01, and *** *p* < 0.001 vs. control.

To evaluate the effect of *B. congestiflora* extract alone on vascular response, cumulative doses were added to the bath in intact and endothelial-denuded rat aortas. As shown in Figure 2A, extract concentrations of 100 and 1000 μg/mL caused significant vascular contraction in intact rat aortas. In Figure 2B, the data showed an increase in contraction of intact rat aortas (control): 41 ± 13% at 100 μg/mL and 71 ± 9% at 1000 μg/mL. In the denuded-endothelium rat aortas (denuded), the same concentrations produced higher contractions: 115 ± 11% at 100 μg/mL and 134 ± 18% at 1000 μg/mL. Vascular contraction of *B. congestiflora* showed an EC_50_ of 96 μg/mL in intact rat aortas (control) and an EC_50_ of 44 μg/mL in denuded-endothelium rat aortas (denuded).

We unexpectedly found that *B. congestiflora* increased vascular tone. To investigate this, we conducted experiments to determine the extract’s mechanism of action. Our protocol involved contracting tissue with 1000 µg/mL of the extract, both with and without nimodipine (a voltage-gated calcium channel blocker). This allowed us to assess whether the extract’s contractile effect relied on extracellular calcium. Additionally, we used caffeine to release calcium from the sarcoplasmic reticulum, exploring its potential role in inducing the extract’s contraction. As shown in Figure 2C,D, preincubation with caffeine reduced the contractile response of *B. congestiflora* in a dose-dependent manner: 104 ± 8% in the control group, 23 ± 9% (*p* < 0.01) with 10 mM caffeine, and 4 ± 0% (*p* < 0.001) with 20 mM caffeine. Interestingly, the reduction in contraction with caffeine was greater than that with nimodipine: 57 ± 13% (*p* < 0.05).

### 2.3. B. congestiflora Extract Produces Endothelium-Dependent Vasorelaxation, via NO, in Rat Aortas

**Figure 3:**
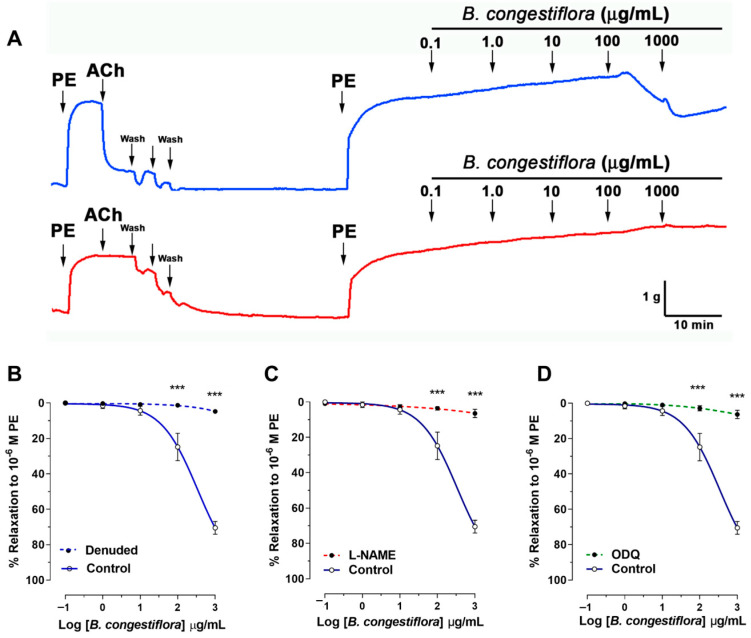
Endothelium-dependent effects of *B. congestiflora* on vascular function. (**A**) Representative original trace showing the vascular relaxation of the hydroalcoholic extract of *B. congestiflora* (0.1 to 1000 mg/mL) in intact aortic rings with endothelium (blue line). (**B**) Concentration-dependent relaxation to *B. congestiflora* in intact (control), endothelium-denuded (denuded; red line), (**C**) L-NAME-treated (10^−4^ M), or (**D**) ODQ-treated (10^−6^ M) aortic rings. Data in this figure are presented as mean ± SEM (*n* = 5). *** *p* < 0.001 vs. control.

The vasorelaxation effect of the hydroalcoholic extract of *B. congestiflora* was assessed using precontracted aortic rings. In these rings, which were precontracted with 10^−6^ M phenylephrine, the control group exhibited a dose-dependent vasorelaxation, achieving 25 ± 8% relaxation at 100 μg/mL and 70 ± 4% at 1000 μg/mL (Figure 3A). However, this relaxant effect was significantly impaired when the vascular endothelium was removed (Figure 3A), or when the tissues were preincubated with either the nonselective Nitric Oxide Synthase (NOS) inhibitor N^ω^-nitro-L-arginine methyl ester (L-NAME; 10^−4^ M; Figure 3B) or the soluble guanylyl cyclase inhibitor 1H-oxadiazolo[3-a]quinoxalin-1-one (ODQ; 10^−6^ M; Figure 3C). Specifically, following endothelium removal, vasorelaxation was dramatically reduced to 1 ± 1% (*p* < 0.001) at 100 μg/mL and 5 ± 1% at 1000 μg/mL. Similarly, in the presence of L-NAME, the vasorelaxation was only 3 ± 1% (*p* < 0.001) at 100 μg/mL and 6 ± 2% at 1000 μg/mL. With ODQ preincubation, the effect was 3 ± 1% at 100 μg/mL, although it reached 61 ± 2% at the higher dose of 1000 μg/mL. These findings collectively indicate that the endothelium and specific signaling pathways are critical mediators of the *B. congestiflora* extract’s vasorelaxant properties.

### 2.4. Enzyme-Inhibitory Properties

Several important enzymes related to CNCD are analyzed using the hydroethanolic extract of the endemic fruits of *B. congestiflora*. The results are shown in Table 1. Enzymes such as glucosidases, amylases, and lipases contribute to metabolic syndrome. In particular, α-glucosidase and α-amylase hydrolyze glycogen and starch to release glucose, linking them to diabetes as well as infections and cancer [21]. Blocking these two enzymes slows the breakdown and absorption of carbohydrates, thereby mitigating post-meal blood sugar spikes [22]. In our work, BE extract inhibits α-amylase and α-glucosidase, and is shown to be active against acetylcholinesterase (AChE) and butyrylcholinesterase (BuChE). Cholinesterase enzymes catalyze the breakdown of acetylcholine into choline and acetate, a key factor in Alzheimer’s disease. Inhibiting these enzymes, sometimes with phenolic compounds, helps maintain acetylcholine levels, which strengthens cholinergic transmission and alleviates the symptoms of Alzheimer’s disease [23]. Berberine, the main alkaloid from leaves of *B. vulgaris*, is shown to inhibit cholinesterase enzymes [24]. In this study, some notable results are those of acetylcholinesterase (IC_50_: 7.33 ± 0.22 μg/mL), which is several times less active than the standard galantamine (IC_50_: 0.40 + 0.02 μg/mL) and butyrylcholinesterase (IC_50_: 19.45 ± 0.32 μg/mL). On the other hand, the inhibitory effect of *Berberis vulgaris* crude extract on α-glucosidase is shown to be more potent than that of berberine chloride [24]; however, our inhibition of α-amylase is moderate (IC_50_: 27.21 ± 0.03 μg/mL) and inhibition of α-glucosidase is high (IC_50_: 243.23 ± 0.3 μg/mL) compared to acarbose, being an extract mainly chemically characterized by anthocyanins. Chilean native *Azara serrata* anthocyanin-enriched extract shows an IC_50_ of 371.6 and 7.23 μg/mL in glucosidase and amylase inhibition tests, respectively, and IC_50_ 3.92 μg/mL in inhibition of AChE that can be attributed in part to the content of active anthocyanins [25]. The observed cholinesterase inhibitory activity suggests the potential for discovering a naturally occurring compound that could serve as a promising candidate for future therapeutic development (Table 1). For future studies, it is important to test new concentrations to validate the potential of the *B. congestiflora* fruit extract (BE) in relation to the inhibition of the cholinesterase, α-glucosidase, and α-amylase enzymes; it is also important to isolate its major anthocyanin compounds, which may show better inhibitory activity. Conversely, the extract exhibits notable inhibition of cholinesterase enzymes—particularly acetylcholinesterase—highlighting its potential to modulate cholinergic signaling in disorders such as Alzheimer’s disease. Nonetheless, further work is required to isolate and characterize its active constituents and to fully elucidate their mechanisms of action. Likewise, exploring additional bioactivities of the *B. congestiflora* extracts, such as their anti-cancer effects, will be crucial to confirm its broader therapeutic potential.

### 2.5. Analysis of the Phenolic and Anthocyanin Profile of BE

#### 2.5.1. Chromatographic Analysis

**Figure 4:**
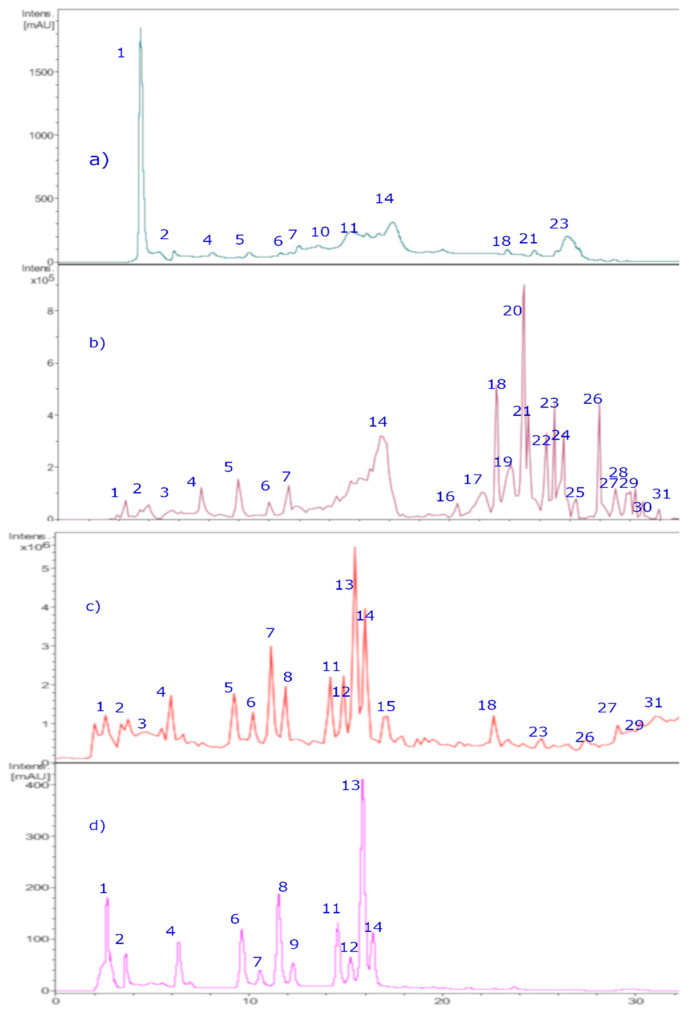
HPLC-DAD chromatograms of *Michay* fruit extracts. (**a**) Chromatograms at 280 nm. (**b**) TIC (total ion current, negative mode). (**c**) Chromatograms at 520 nm. (**d**) TIC (total ion current, positive mode).

**Figure 5:**
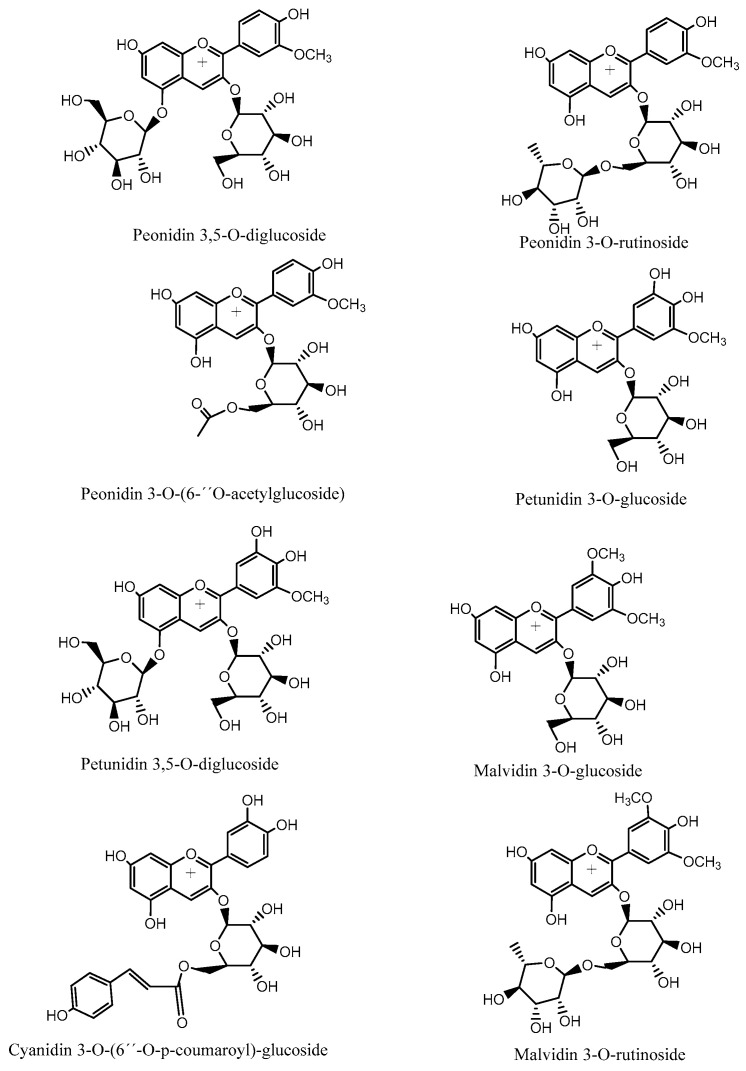
Structures of representative anthocyanin compounds identified in Chilean *Michay* berries.

The chromatographic analysis in this study was performed using UHPLC-DAD and HPLC-ESI (+)-MS^n^ (Figure 4). The main class of detected compounds was the anthocyanins. Anthocyanins are a class of natural pigments that are known for their colors, ranging from blue to red and purple, in relation to fruits, vegetables, and flowers, as well as for the bioactivity that some of them possess. Anthocyanins have characteristic absorption spectra, so the wavelengths of the maxima of each compound were determined, while molecular ions were determined using ESI (+)-MS^n^ measurements. According to this, thirty-one peaks (Figure 4 and Figure 5, Table 2) were tentatively identified for the first time in the *BE* extract using positive and negative ESI modes, as shown in Table 2.

##### Anthocyanins

**Figure 6:**
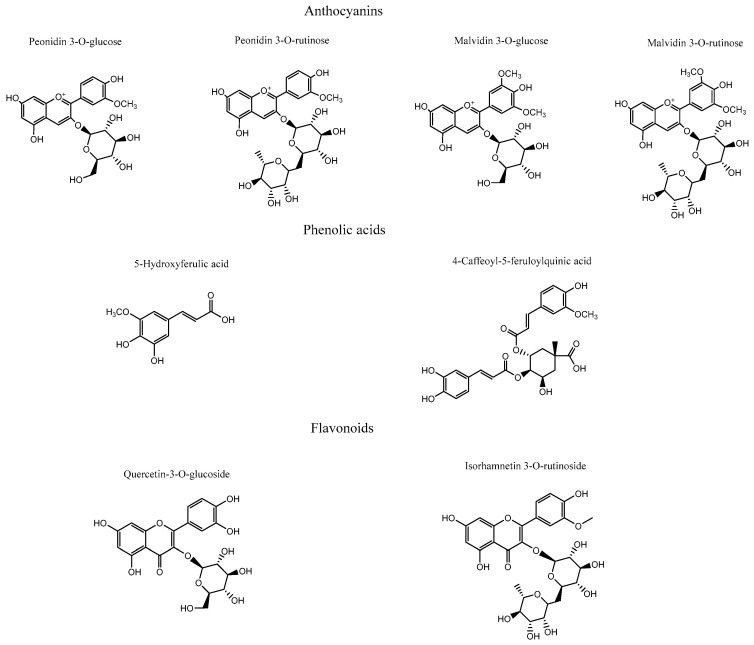
Compounds subjected to docking assays in the corresponding catalytic sites of acetylcholinesterase, butyrylcholinesterase, glucosidase, and α-amylase.

Nine known anthocyanins were identified in *Michay* fruits (peaks 4, 5, 6, 7, 8, 11, 12, 13, 14, and 15 in Figure 6) with molecular ions in positive mode at *m*/*z* 609, 479, 609, 465, 505, 625, 639, 449, 493, and 331 and showing characteristic MS^2^ ions at *m*/*z* 463 (MS^3^ ions at *m*/*z* 301, 286, and 147), 303 (MS^3^ ion at *m*/*z* 257), 301 (MS^3^ ion at *m*/*z* 286), 317 (MS^3^ ion at *m*/*z* 302), 331 (MS^3^ ion at *m*/*z* 299 and 179), and 299 (MS^3^ ion at *m*/*z* 179), respectively, corresponding to petunidin 3-O-glucoside (λ max: 275–343 sh-512 nm), peonidin 3-O-rutinoside (λ max: 268, 357 sh, 503 nm), delphinidin 3-O-glucoside (λ max: 275–341 sh-512 nm), peonidin 3-O-glucoside, (λ max: 268–347 sh-512 nm), peonidin 3-O-[6″-O-(acetyl)]-glucoside, peonidin 3,5-di-O-glucoside, malvidin 3-O-rutinoside, malvidin 3-O-glucoside, and malvidin, respectively. Other minor compounds were peaks 1–3 with pseudomolecular cations at *m*/*z* 611, 595, and 641, and thus were identified as cyanidin 3,5-O-diglucoside, cyanidin 3-O-[6″-O-(p-coumaroyl)] glucoside, and petunidin 3,5-O-diglucoside, respectively. Some of the identities were corroborated by co-elutions with some standard anthocyanins in our laboratory and literature data [26,27].

##### Phenolic Acids

Peaks 9, 10, 25–27, and 30 were identified as phenolic acids according to their UV spectra (Table 2). Peaks 9 and 10 with molecular anions at *m*/*z* 515 and *m*/*z* 353 showing an MS daughter ion at *m*/*z* 191 (quinic acid) were related to the phenolics: diccafeoyl quinic acid and caffeoyl quinic acid, respectively [28]. Peak 25 with a pseudomolecular ion at *m*/*z* 529 and showing an MS^2^ ion at *m*/*z* 367 was identified as 4-Caffeoyl-5-feruloylquinic acid [29], while peak 26 was identified as the lignan 8-hydroxypinoresinol [30]. Peak 27 with a pseudomolecular ion at *m*/*z* 371 and a diagnostic (2M-H-) ion at *m*/*z* 742 was identified as 5-hydroxyferulic acid [31]. Peak 30 was identified as unknown compound, but matched the structure of caffeoyl-2-hydroxyethane-1,1,2-tricarboxylic acid [32].

##### Flavonoids

Peaks 16–24, 28, and 29 were determined as flavonoid derivatives according to UV spectra and MS^n^ peaks (Table 2). Peak 16 with a parent molecular ion at *m*/*z* 463, showing two UV λ max at 255 and 355 nm, characteristic of flavonols and daughter ions at *m*/*z* 301, 179, and 151, was identified as quercetin-3-O-β-D-galactopyranoside (hyperoside), while peak 17 was identified as Quercetin-3-O-([6″-O-(acetyl)]-glucopyranoside. Peak 18 was identified as isorhamnetin-3-O-β-D-glucopyranoside while peak 19 with a molecular anion at *m*/*z* 623 producing an MS^2^ ion at *m*/*z* 315 was identified as isorhamnetin 3-O-rutinoside (narcissin) [33], while peak 24 with a parent ion at 519 and daughter ions at *m*/*z* 477 and 315 was identified as isorhamnetin-3-O-([6″-O-(acetyl)]-glucoside) [34]. Peak 20 was identified as quercetin-3-O-glucoside and peak 21 as rutin. Peak 22 was identified as luteolin 7-O-β-D-glucopyranoside [35], and peak 23 as phloretin 3′,5′-Di-C-glucoside [36]. Finally, peaks 28 and 29 were identified as the isomers isorhamnetin and rhamnetin, and peak 31 as quercetin, respectively.

#### 2.5.2. Docking Calculations Results

To more accurately assess and elucidate the inhibition results for acetylcholinesterase, butyrylcholinesterase, glucosidase, and α-amylase, we considered the data shown in Table 2. Thus, four anthocyanins (peonidin 3-O-glucoside, peonidin 3-O-rutinoside, malvidin 3-O-glucoside, and malvidin 3-O-rutinoside), two phenolic acids (5-hydroxyferulic acid and 4-caffeoyl-5-feruloylquinic acid), and two flavonoids (isorhamnetin 3-O-rutinoside and isorhamnetin-3-O-glucoside), as shown in Figure 6, along with the known inhibitors galantamine and acarbose, were selected to perform docking analyses. Each docking assay was performed within the catalytic site of each enzyme to evaluate the docking energy descriptor and the molecular interactions between the active-site residues and the selected metabolites. This step sought to rationalize the inhibitory activities of the extract toward the enzymes. Table 3 shows the binding energies of the selected anthocyanins, phenolic acids, and flavonoids.

##### Docking Results for Acetylcholinesterase (TcAChE)

**Figure 7:**
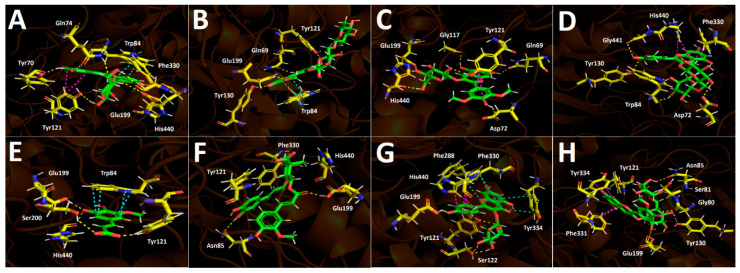
This presents the predicted intermolecular interactions between selected compounds from the *Berberis congestiflora* Gay (BE) ethanolic extract and the residues of the Torpedo californica acetylcholinesterase (TcAChE) catalytic site. Yellow dotted lines indicate hydrogen-bond interactions, cyan dotted lines represent π π–π π interactions, magenta dotted lines correspond to T-shaped interactions, and red dotted lines denote π π–cation interactions. Panel (**A**) shows peonidin 3-O-glucoside in the catalytic site, panel (**B**) peonidin 3-O-rutinoside, panel (**C**) malvidin 3-O-glucoside, and panel (**D**) malvidin 3-O-rutinoside. Panel (**E**) illustrates 5-hydroxyferulic acid in the catalytic site, panel (**F**) 4-Caffeoyl-5-feruloylquinic acid, panel (**G**) quercetin-3-O-glucoside, and panel (**H**) isorhamnetin 3-O-rutinoside bound within the catalytic site.

The ethanolic extract of *Berberis congestiflora* Gay (BE) exhibited moderate acetylcholinesterase inhibition (IC_50_: 7.33 ± 0.22 µg/mL), which is overall quite reasonable, being only one order of magnitude higher than that of galantamine. The energies reported in Table 3 indicate that the compounds peonidin 3-O-glucose, peonidin 3-O-rutinose, malvidin 3-O-glucose, quercetin-3-O-glucoside, and isorhamnetin 3-O-rutinoside possess favorable binding energies. This outcome could be attributed to the fact that the whole extract is a mixture of compounds that may compete for the catalytic site of the enzyme, thereby interfering with a more specific inhibition. Thus, it is possible that measuring the inhibitory activity of each isolated derivative would reveal strong acetylcholinesterase inhibition. Overall, all anthocyanins exhibited optimal binding energies superior to that of galantamine, except for malvidin 3-O-rutinoside, which showed a value of −11.632 kcal/mol. The anthocyanins peonidin 3-O-glucoside, peonidin 3-O-rutinoside, and malvidin 3-O-glucoside displayed favorable binding profiles within the catalytic site, suggesting a relevant contribution to the overall inhibition pattern observed in the docking analysis. All anthocyanins were stabilized within the acetylcholinesterase catalytic site mainly through hydrogen-bond interactions, but they also exhibited π–π and T-shaped interactions. Peonidin 3-O-glucoside formed seven hydrogen bonds through the oxygenated functions of its glucose moiety and the methoxyphenylchromenylium core of its structure with the amino acids Tyr70, Gln74, Tyr121, Glu199, Phe330, and His440. In addition, peonidin 3-O-glucoside formed a π–π interaction with Trp84 and a T-shaped interaction with Tyr121 (Figure 7A). Peonidin 3-O-rutinoside arranged within the catalytic site in a similar manner to peonidin 3-O-glucoside and therefore also formed hydrogen bonds with the amino acids Gln69, Tyr130, Glu199, and Tyr121. Likewise, through its methoxyphenylchromenylium core, it formed a π–π interaction with Trp84 and a T-shaped interaction with Tyr121 (Figure 7B). The anthocyanin malvidin 3-O-glucoside, like the previous two compounds, also formed hydrogen-bond interactions with Gln69, Asp72, Gly117, Tyr121, Glu199, and His440, as well as a T-shaped interaction with Tyr121 (Figure 7C).

By contrast, the anthocyanin malvidin 3-O-rutinoside, which showed a binding energy of −11.632 kcal/mol and an energy profile quite similar to that of galantamine, formed only four hydrogen bonds with Asp72, Trp84, Tyr130, and Gly441, along with a T-shaped interaction with His440 (Figure 7D). The evaluated phenolic acids, 5-hydroxyferulic acid and 4-caffeoyl-5-feruloylquinic acid, did not exhibit favorable binding energies (−8.647 kcal/mol and −10.068 kcal/mol, respectively). The energy values for both derivatives were lower than those of the anthocyanins discussed above, as well as that of galantamine (Table 3). To assess the relevance of the sugar moieties, 5-hydroxyferulic acid was selected as a compound lacking a sugar portion, whereas 4-caffeoyl-5-feruloylquinic acid contained a carbohydroxy framework. In this regard, the latter showed a better interaction profile within the acetylcholinesterase catalytic site. Specifically, 5-hydroxyferulic acid formed four hydrogen bonds with Tyr121, Glu199, Ser200, and His440, as well as a T-shaped interaction with Trp84 (Figure 7E), whereas 4-caffeoyl-5-feruloylquinic acid formed only two hydrogen bonds with Asn85 and Glu199 (Figure 7F). The flavonoids quercetin-3-O-glucoside and isorhamnetin 3-O-rutinoside also showed favorable energy profiles (Table 3), although not superior to those of the anthocyanins. Their main interactions within the catalytic site of the enzyme were hydrogen bonds. In particular, quercetin-3-O-glucoside established four hydrogen bonds with Tyr121, Ser122, Glu199, and Phe288; two π–π interactions with Phe330 and Tyr334; and one T-shaped interaction with His440 (Figure 7G). Isorhamnetin 3-O-rutinoside formed hydrogen bonds with the amino acids Gly80, Ser81, Asn85, Tyr121, Tyr130, and Glu199, as well as two T-shaped interactions with Phe331 and Tyr334 (Figure 7H).

##### Docking Results for Butyrylcholinesterase (hBuChE)

**Figure 8:**
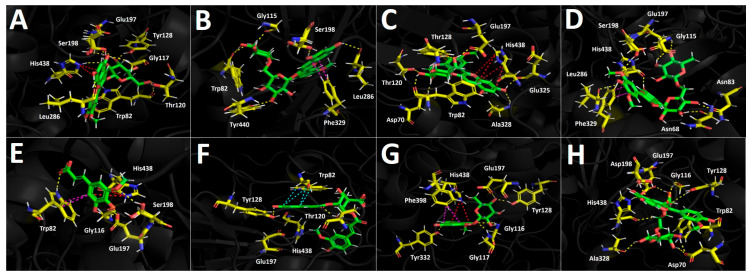
This shows the predicted intermolecular interactions between selected compounds from the *Berberis congestiflora* Gay (BE) ethanolic extract and the residues of the human butyrylcholinesterase (hBuChE) catalytic site. Yellow dotted lines indicate hydrogen-bond interactions, cyan dotted lines represent π π–π π interactions, and red dotted lines correspond to π π–cation interactions. Panel (**A**) depicts peonidin 3-O-glucoside in the catalytic site, panel (**B**) peonidin 3-O-rutinoside, panel (**C**) malvidin 3-O-glucoside, and panel (**D**) malvidin 3-O-rutinoside within the catalytic pocket. Panel (**E**) illustrates 5-hydroxyferulic acid in the catalytic site, panel (**F**) 4-Caffeoyl-5-feruloylquinic acid, panel (**G**) quercetin-3-O-glucoside, and panel (**H**) isorhamnetin 3-O-rutinoside.

Docking simulations performed on human butyrylcholinesterase (hBuChE) provided insights into the binding behavior of the analyzed metabolites. Overall, the assayed anthocyanin, phenolic acid, and flavonoid derivatives exhibited favorable binding energies (Table 3), except for 5-hydroxyferulic acid, which could be attributed to the absence of a saccharide moiety in its structure (−6.753 kcal/mol). Peonidin 3-O-glucoside, which exhibited a binding energy of −12.461 kcal/mol, established several hydrogen-bond interactions with different residues within the butyrylcholinesterase catalytic site, including the amino acids Trp82, Gly117, Thr120, Tyr128, Glu197, Ser198, and Leu286. It also formed a π–cation interaction with His438, whose imidazole ring was protonated at a physiological pH of 7.4 (Figure 8A). The anthocyanin peonidin 3-O-rutinoside exhibited a favorable binding energy of −13.129 kcal/mol, with its main interactions consisting of hydrogen bonds with Trp82, Gly115, Ser198, Leu286, and Tyr440, along with a T-shaped interaction with Phe329. It is noteworthy that these anthocyanins differed in the glycosidic framework of their structures, which allowed them to fit differently within the catalytic site of the enzyme (Figure 8B). A similar pattern was observed for the analogs malvidin 3-O-glucoside and malvidin 3-O-rutinoside, in which the former anthocyanin displayed a less favorable binding energy compared with the latter (Table 3). In this sense, malvidin 3-O-glucoside formed hydrogen-bond interactions with Asp70, Trp82, Tyr128, Glu197, Glu325, and Ala328, as well as a π–cation interaction with His438 (Figure 8C). Likewise, malvidin 3-O-rutinoside carried out several hydrogen-bond interactions with Asn68, Asn83, Gly115, Glu197, Ser198, Leu286, Tyr332, and His438, as well as a T-shaped interaction with Phe329 (Figure 8D). Given the evidence presented above, anthocyanin derivatives bearing rutinose moieties exhibit a better binding profile toward butyrylcholinesterase and therefore could act as more effective inhibitory agents. The compound 5-Hydroxyferulic acid, which lacks a glucoside moiety, also forms hydrogen-bond interactions through its oxygen functions with Trp82, Gly116, Glu197, and Ser198. Additionally, this derivative exhibits a π–cation interaction with His438 and a T-shaped interaction with Trp82 (Figure 8E). The phenolic acid 4-caffeoyl-5-feruloylquinic acid, which contains a glycosidic framework in its structure, exhibits a lower number of hydrogen-bond interactions (with residues Thr120 and Tyr128) and a π-π interaction with Trp82. Nevertheless, its binding energy is improved due to the number of hydrophobic interactions that the glycosidic framework itself may generate (Figure 8F). In the case of the flavonoids quercetin-3-O-glucoside and isorhamnetin-3-O-rutinoside, the presence of the rutinose framework again improves the docking descriptors. Accordingly, the first derivative, which exhibits an energy of −12.808 kcal/mol, forms hydrogen-bond interactions with Gly116, Gly117, Tyr128, Glu197, Ser198, and Tyr332, as well as a T-shaped interaction with Phe398 and a π-π cation interaction with His438 (Figure 8G). Conversely, the second derivative engages in several hydrogen-bond interactions with residues Asp70, Trp82, Gly116, Tyr128, Glu197, Ser198, Ala328, and His438, thereby stabilizing this compound within the catalytic site of butyrylcholinesterase (Figure 8H).

##### Glucosidase Docking Results

**Figure 9:**
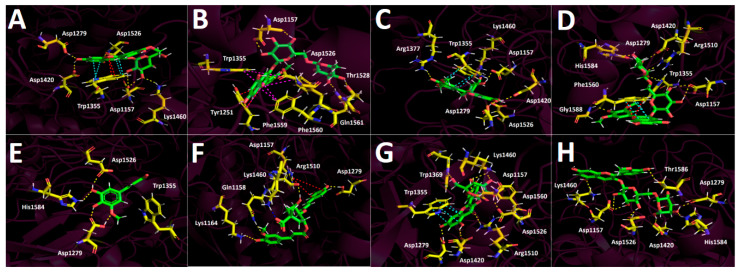
Predicted intermolecular interactions between selected compounds from the ethanolic extract of *Berberis congestiflora* Gay (BE) and the residues within the human glucosidase catalytic site. Yellow dashed lines denote hydrogen bonding interactions, cyan dashed lines indicate π π–π π interactions, magenta dashed lines represent T-shaped interactions, and blue dashed lines correspond to salt bridges. Panel (**A**) shows peonidin 3-O-glucose in the catalytic site; panel (**B**) peonidin 3-O-rutinose in the catalytic site; panel (**C**) malvidin 3-O-glucose in the catalytic site; panel (**D**) malvidin 3-O-rutinose in the catalytic site; panel (**E**) 5-Hydroxyferulic acid in the catalytic site; panel (**F**) 4-Caffeoyl-5-feruloylquinic acid in the catalytic site; panel (**G**) quercetin-3-O-glucoside in the catalytic site; and panel (**H**) isorhamnetin 3-O-rutinoside in the catalytic site.

The results obtained for the glucosidase enzyme reveal distinct differences in how the derivatives accommodate within the catalytic pocket, highlighting the contribution of specific substituents to hydrogen bonding, other noncovalent interactions, and overall stabilization of the enzyme–ligand complex. The binding energies shown in Table 3 indicate that the anthocyanins are the derivatives that would presumably behave as the most effective inhibitors of this enzyme; however, none of them exhibit a more favorable energetic profile than the reference inhibitor acarbose. Peonidin-3-O-glucoside, peonidin-3-O-rutinoside, and malvidin-3-O-glucoside display highly favorable binding energy values (Table 3). These three derivatives, which share the phenylchromenylium framework, are predominantly stabilized by hydrogen-bond interactions, but they also engage in π-π and π–cation interactions. Peonidin-3-O-glucoside engages in hydrogen-bond interactions with Asp1157, Asp1279, Asp1420, Lys1460, and Asp1526, as well as π-π and π–cation interactions with Trp1355 (Figure 9A). Peonidin-3-O-rutinoside likewise forms hydrogen-bond interactions with Asp1157, Asp1526, Thr1528, and Gln1561, as well as a π–cation interaction with Tyr1251 and three T-shaped interactions with Trp1355, Phe1559, and Phe1569 (Figure 9B). The malvidin-3-O-glucoside derivative, which exhibits the best energetic profile (Table 3), shows hydrogen-bond interactions with Asp1157, Asp1279, Arg1377, Asp1420, Asp1526, and Lys1460, along with a π-π interaction with Trp1355 (Figure 9C). Similarly, malvidin-3-O-rutinoside forms eight hydrogen-bond interactions with residues Asp1157, Asp1279, Trp1355, Asp1420, and Arg1510, along with a π-π interaction with Phe1560 and a π–cation interaction with Phe1560 (Figure 9D). The phenolic acid 5-hydroxyferulic acid, due to the nature of its structure, which lacks a carbohydrate core, only engages in hydrogen-bond interactions with Asp1279, Asp1526, and His1584 (Figure 9E), which likely accounts for its binding energy of −8.017 kcal/mol. In contrast, the structural complexity of 4-caffeoyl-5-feruloylquinic acid allows it to fit within the glucosidase catalytic site in a manner that gives rise to hydrogen-bond interactions with Asp1157, Gln1158, Lys1164, and Asp1279, as well as two additional key contacts: a π–cation interaction with Arg1510 and a salt-bridge interaction with Lys1460 (Figure 9F), which ultimately results in a more favorable binding energy (Table 3).

Finally, the flavonoids quercetin-3-O-glucoside and isorhamnetin-3-O-rutinoside present in the *Berberis congestiflora* Gay (BE) ethanolic extract may be considered good candidates as potential glucosidase inhibitors; however, they do not reach the favorable profiles observed for the aforementioned anthocyanins (peonidin-3-O-glucoside, peonidin-3-O-rutinoside, and malvidin-3-O-glucoside). In this sense, the main interactions of quercetin-3-O-glucoside are hydrogen-bond interactions with Asp1157, Asp1279, Trp1369, Asp1420, Lys1460, Arg1510, and Asp1526, as well as a π-π interaction with Trp1355 (Figure 9G). For isorhamnetin-3-O-rutinoside, the interactions observed are predominantly hydrogen-bond interactions, with no other types of noncovalent contacts detected. This could account for the weaker binding energy of this derivative compared with quercetin-3-O-glucoside (Table 3). The amino acids involved in the hydrogen-bond interactions of isorhamnetin-3-O-rutinoside are Asp1157, Asp1279, Asp1420, Lys1460, Asp1526, His1584, and Thr1586 (Figure 9H).

##### α-Amylase Docking Results

Docking analysis performed against α-amylase provides insight into the structural determinants governing ligand accommodation within the catalytic pocket. As shown in Table 3, the results reveal distinct differences in binding energies among the derivatives, highlighting the molecular basis for interpreting their potential inhibitory effects on alpha-amylase activity. Although the anthocyanins exhibit binding energies higher (less favorable) than that of the reference inhibitor acarbose in the α-amylase docking assays, their values can still be considered moderately acceptable overall. Notably, malvidin-3-O-rutinoside is the only derivative that displays the most favorable energetic profile, with a binding energy of −14.120 kcal/mol.

**Figure 10:**
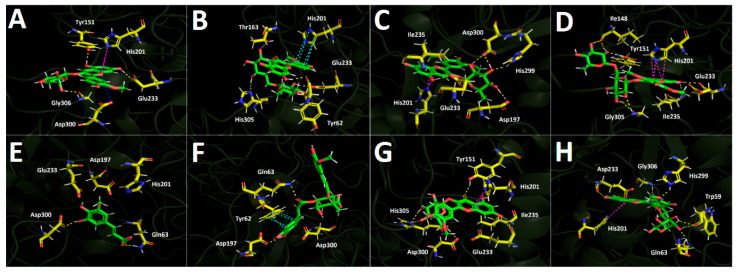
Predicted intermolecular interactions of selected compounds in *Berberis congestiflora* Gay (BE) ethanolic extract and the residues of the human pancreatic α-amylase catalytic site. Yellow dotted lines indicate hydrogen-bond interactions, magenta dotted lines represent T-shaped interactions, and blue dotted lines represent salt bridges. Panel (**A**) shows peonidin 3-O-glucoside in the catalytic site; panel (**B**) peonidin 3-O-rutinoside in the catalytic site; panel (**C**) malvidin 3-O-glucoside in the catalytic site; panel (**D**) malvidin 3-O-rutinoside in the catalytic site; panel (**E**) 5-Hydroxyferulic acid in the catalytic site; panel (**F**) 4-Caffeoyl-5-feruloylquinic acid in the catalytic site; panel (**G**) quercetin-3-O-glucoside in the catalytic site; and panel (**H**) isorhamnetin 3-O-rutinoside in the catalytic site.

The derivatives bearing a glucose moiety in their structures exhibit poorer binding energies compared with those containing the rutinose core. In this regard, although peonidin-3-O-glucoside engages in a salt-bridge interaction with Asp300 and a T-shaped interaction with His201, these appear to be insufficient, even when considering the hydrogen-bond interactions it forms with Tyr151, Glu233, Asp300, and Gly306 (Figure 10A). Peonidin-3-O-rutinoside accommodates itself within the α-amylase catalytic site in a distinct manner compared with peonidin-3-O-glucoside, which likely accounts for its more favorable binding energy pattern. This derivative establishes hydrogen-bond interactions with Tyr62, Thr163, Glu233, and His305, as well as a π-π interaction between the imidazole ring of His201 and the phenyl ring of the phenylchromenylium moiety of this anthocyanin (Figure 10B). Malvidin-3-O-glucoside likewise engages in an interaction with His201, specifically a T-shaped contact involving the imidazole ring of the amino acid. Furthermore, the residues involved in hydrogen-bond interactions with this derivative are Asp197, Glu233, Ile235, His299, and Asp300 (Figure 10C). Malvidin-3-O-rutinoside also forms a T-shaped interaction with His201; as expected, it additionally establishes hydrogen-bond interactions with Ile148, Tyr151, Glu233, Ile235, and Gly306, which contribute to stabilizing this compound within the catalytic site (Figure 10D). The phenolic acids 5-hydroxyferulic acid and 4-Caffeoyl-5-feruloylquinic acid, similar to what is observed for the other enzymes, are the derivatives that display the most unfavorable binding energy performance (Table 3). This outcome could be attributed to the limited number of interactions established by each of the derivatives. Specifically, 5-hydroxyferulic acid engages in only two hydrogen-bond interactions with Gln63 and Asp300 (Figure 10E), whereas 4-Caffeoyl-5-feruloylquinic acid also forms two hydrogen-bond interactions with Gln63 and Asp197, in addition to a π-π interaction with Tyr62 (Figure 10F). Regarding the two flavonoids assayed, quercetin-3-O-glucoside forms hydrogen-bond interactions with Tyr151, Glu233, Ile235, Asp300, and His305, along with a T-shaped contact involving His201 within the α-amylase catalytic site (Figure 10G). In contrast, isorhamnetin-3-O-rutinoside engages in hydrogen-bond interactions with Trp59, Gln63, Glu233, His299, and Gly306, while similarly establishing a T-shaped interaction with His201 (Figure 10H). These interaction patterns suggest that both flavonoid derivatives can achieve a stable binding pose, as reflected in the binding energies reported in Table 3.

## 3. Materials and Methods

### 3.1. Chemicals, Reagents, and Materials

Ethyl acetate ultrapure water, ethanol, ascorbic acid, Folin–Ciocalteu reagent, AlCl_3_, FeCl_3_, gallic acid, magnesium metal, quercetin, Fe_2_SO_4_, dimethyl sulfoxide, 2,4,6-tripyridyl-s-triazine (TPTZ), 2,2′-azo-bis(2-amidinopropane dichlorohydrate), 2,2-diphenyl-1-picrylhydrazyl (DPPH), and other analytical-grade solvents were supplied by Merck (Merck, Darmstadt, Germany). Trolox, β β-apo-8′-carotenal, and DMSO (purity > 95%) were obtained from Sigma-Aldrich Chem. Co. (St. Louis, MO, USA), Phytolab GmbH & Co. KG (Vestenbergsgreuth, Germany), or Extrasynthese (Genay, France). Acetylcholinesterase (TcAChE, EC 3.1.1.7), butyrylcholinesterase (hBuChE, EC 3.1.1.8), L-glutamine, 4-nitrophenyldodecanoate, phosphate buffer, dinitrosalicylic acid, trichloroacetic acid were supplied by Merck (Merck, Darmstadt, Germany), fetal calf serum (FCS, Gibco, Grand Island, NY, USA), α-amylase, α-glucosidase, standard p-nitrophenyl-D-glucopyranoside, acarbose, anhydrous sodium sulfate, nsodium persulfate, sodium carbonate, ferrous sulfate, absolute ethanol (99.5%),sodium acetate, and the HPLC standard with purity higher than 95% (flavonoids, phenolic acids, and anthocyanins), were purchased from Sigma-Aldrich Chem. Co. (St. Louis, MO, USA), Biopurify (Chengdu, China) or Xtrasynthese (Genay, France). Double-deionized water was produced using a Nanopure system (Werner GmbH, Leverkusen, Germany). Disodium hydrogen phosphate dihydrate (≥99.0%, p.a.) and citric acid (≥99.5%, p.a.) were provided by Carl Roth GmbH & Co. KG (Karlsruhe, Germany). The solvents used for UHPLC–DAD analyses were water (LC–MS grade) and methanol (UHPLC–MS grade), purchased from TH. Geyer GmbH & Co. KG (Renningen, Germany). L-phenylephrine hydrochloride, acetylcholine chloride, indomethacin, sodium nitroprusside dihydrate (SNP), plus N^ω^-nitro-L-arginine methyl ester (L-NAME) were obtained from Sigma (St. Louis, MO, USA), and 1H-[1,2,4]oxadiazolo[4,3-a]quinoxalin-1-one (ODQ) was provided by Cayman Chemical (Ann Arbor, MI, USA). Phenylephrine and acetylcholine were added to distilled water, whereas the remaining drugs were prepared in dimethyl sulfoxide; in rat experiments, the final concentration of vehicle did not exceed 0.1% (*v*/*v*).

### 3.2. Plant Material

The mature fruits of *Berberis congestiflora* (Figure 1) plant were collected during February 2017 in the farm called “Las Hortensias” located in the Region of Araucanía, Chile. Subsequently, the fruits were lyophilized (Labconco Freeze Dry Systems, Model 7670541 2.5 Liter Palo Alto, CA, USA, −50 °C, vacuum 0.13 barr) and stored at −80 °C in an ultrafreezer (Haier, biomedical model Dw86L388A, Qingdao, China). Plant and fruits were identified by the botanist Alicia Marticorena from Universidad de Concepcion, Chile.

### 3.3. Berry Extract Preparation

Dried and ground fruits (200 g) were macerated in a mixture EtOH: H_2_O (1:1 *v*:*v*) for 1 h with sonication (three times). Subsequently, the extract mixture was combined and filtered on Whatman N° 4 paper and subjected to a rotary evaporator at a temperature of 50 °C to evaporate the ethanol. The extract obtained was lyophilized and stored at 4 °C until its use (18.3 g).

### 3.4. Chemical Analyses

#### 3.4.1. Total Polyphenol, Anthocyanin, and Flavonoid Quantification

Standardized protocols were employed to determine the total phenolic content (TPC), total anthocyanins (TAC), and total flavonoid content (TFC). Absorbance was recorded in a multiplate reader (Agilent BioTek Synergy HTX Multi-Mode Microplate Reader, Billerica, MA, USA). The calibration curves for TPC were created using gallic acid and for TFC using a quercetin standard [25,37]. The phenolic compound content and flavone content were expressed as mg of gallic acid (mg GAE/g) and mg of quercetin per g of sample (mg QE/g), respectively. The assessment of total anthocyanin content (TAC) was carried out by the pH differential method according to AOAC as described previously using cyanidin 3-O-glucoside equivalents (mg c3g/g) [17].

#### 3.4.2. HPLC Analysis and Mass Spectrometric Conditions

A Merck–Hitachi LaChrom system (Tokyo, Japan), equipped with an L-7100 pump, an L-7455 UV diode array detector, a D-7000 chromato-integrator, and a column compartment, was employed for the analyses. Separation was carried out on a Purospher Star C18 column (250 mm × 5 mm, 4.6 mm i.d., Merck, Germany). The mobile phase consisted of solvent A (10% formic acid in water) and solvent B (acetonitrile). The gradient elution for HPLC–DAD and HPLC–ESI–MS started with 90% A for the first 4 min, followed by a linear change to 75% A over 25 min, and then a linear return to the initial conditions over an additional 15 min. The flow rate was maintained at 1 mL/min, and the column temperature was kept at 25 °C. Detection was performed at 520 nm for anthocyanins and 320–280 nm for other compounds, while UV spectra from 200 to 600 nm were recorded for peak characterization. For HPLC–ESI–MS analysis, an Esquire 4000 ion trap mass spectrometer (Bruker Daltoniks, Bremen, Germany) was coupled to an Agilent 1100 HPLC (Agilent, Waldbronn, Germany) system via an ESI interface. Full-scan mass spectra were acquired from *m*/*z* 150 to 2000 u, using positive-ion mode for anthocyanins and negative-ion mode for the remaining compounds. High-purity nitrogen served as nebulizing gas at 27.5 psi, 350 °C, and a flow rate of 8 L/min. In negative-ion mode, the MS parameters were electrospray needle, 4000 V; end plate offset, −500 V; skimmer 1, −56.0 V; skimmer 2, −6.0 V; and capillary exit offset, −84.6 V. In positive-ion mode, the settings were as follows: electrospray needle, 4000 V; end plate offset, −500 V; skimmer 1, 56.0 V; skimmer 2, 6.0 V; capillary exit offset, 84.6 V; and capillary exit, 140.6 V. Collisionally induced dissociation (CID) MS/MS spectra were recorded with a fragmentation amplitude of 1.00 V, using ultrahigh-purity helium as the collision gas.

### 3.5. Antioxidant Activity

The free radical scavenging ability and overall antioxidant capacity of the different extracts were evaluated using spectrophotometric methods with a microplate reader (Synergy HTX, Billerica, MA, USA).

#### 3.5.1. Oxygen Radical Absorbance Capacity (ORAC) Assay

A detailed protocol for this test can be found in the Supplementary Material. The ORAC assay assesses radical scavenging activity by exposing the samples to 2,2′-azobis(2-amidinopropane) dihydrochloride (AAPH). Fluorescence is monitored at an excitation wavelength of 485 nm and an emission wavelength of 530 nm, and the responses are quantified using a Trolox calibration curve. The results are reported as μmol Trolox per gram of dry fruit [25]. A more detailed description of the assay procedure is provided in the Supplementary Material.

#### 3.5.2. Ferric Reducing Antioxidant Power (FRAP) Assay

This method quantifies antioxidant capacity by reducing the ferric–TPTZ complex (Fe^3+^–TPTZ) to its ferrous form (Fe^2+^–TPTZ), which imparts a blue color. Absorbance is read at 593 nm, and results are calculated against a Trolox calibration curve, then reported as μmol Trolox per gram of dry fruit [25]. A full protocol is available in the Supplementary Material.

#### 3.5.3. DPPH Scavenging Activity

Antioxidant activity is determined by the decolorization of the DPPH radical as it accepts protons, yielding a colorless solution. The decrease in absorbance at 515 nm is measured spectrophotometrically and compared to a gallic acid standard curve. Results are expressed as the IC_50_ (μg/mL)—the concentration required to inhibit 50% of the DPPH radical [38]. Detailed steps appear in the Supplementary Material.

#### 3.5.4. ABTS Scavenging Activity

The assay employed ABTS (2,2′-azinobis-(3-ethylbenzothiazoline-6-sulfonic acid); Sigma Aldrich, St. Louis, MO, USA). Absorbance readings were taken at 1 and 6 min after initiating the reaction, and percent decolorization was calculated [19]. All measurements were performed in triplicate, and the IC_50_—the concentration required to neutralize 50% of the ABTS radicals—was determined. A full protocol is provided in the Supplementary Material.

##### Vascular Reactivity Experiments

All animal procedures were approved by the Institutional Bioethics Committee of the Universidad de Antofagasta (CEIC-UA 135/2018) and were in accordance with the Guide for the Care and Use of Laboratory Animals (115-2018). Male Sprague-Dawley rats (180–200 g) were killed by cervical dislocation and the thoracic aorta was removed, placed in a cold physiological Krebs-Ringer Bicarbonate (KRB) solution (in mM: 4.2 KCl, 1.19 KH_2_PO_4_, 120.0 NaCl, 25.0 Na_2_HCO_3_, 1.2 MgSO_4_, 1.3 CaCl_2_, 5.0 D-Glucose, pH 7.4), gassed with 95% O_2_ and 5% CO_2_, and maintained at 37 °C. Fat and connective tissue were removed, and the thoracic aorta was cut into equal-sized ring segments of 2–3 mm in length. In some cases, the endothelium was removed by gentle rubbing of the luminal surface with a piece of cotton. Each ring was suspended in an organ bath containing 5 mL of KRB solution and then allowed to equilibrate for a period of 60 min under 1 g of resting. Isometric tension was recorded using an isometric force transducer (Radnoti, Monrovia, CA, USA) connected to an acquisition system (PowerLab 8/35, ADInstruments, Colorado Springs, CO, USA).

### 3.6. Aortic Experimental Protocols

After a 60 min of equilibration period, all aortic rings were initially exposed twice to 60 mM KCl. Tissues that failed to produce a 1 g increase in tension were rejected. Endothelial integrity was tested by the action of acetylcholine (10^−5^ M) in segments previously contracted with phenylephrine (10^−6^ M). A relaxation equal to or greater than 70% was considered as demonstrative of the functional integrity of the endothelium. After a washout period, increasing concentrations of *B. congestiflora* extract (0.1–1000 µg/mL) were applied, and concentration–response curves were obtained in aortic rings previously contracted with phenylephrine (10^−6^ M). In some rings, the effects of the following drugs were evaluated: (1) the nonselective NOS inhibitor L-NAME (10^−4^ M) and (2) the soluble guanylyl cyclase inhibitor ODQ (10^−6^ M). These drugs were added 30 min before the experiments.

### 3.7. Enzymatic Inhibitory Activity

#### 3.7.1. Acetylcholinesterase and Butyrylcholinesterase Inhibition Assays

The inhibitory activity of the cholinesterase enzymes was evaluated as described previously [37]. Briefly, a solution with 5-dithio-bis (2-nitrobenzoic acid) (DTNB) was prepared in Tris-HCl buffer (pH 8.0) containing 0.02 M MgCl_2_ and 0.1 M NaCl. Then, the hydroethanolic extract of Michay (50 μL, 2 μg/mL) was mixed in a 96-well microplate with 125 μL of DTNB solution, acetylcholinesterase (TcAChE), or butyrylcholinesterase (hBuChE) (25 μL). It was dissolved in Tris-HCl buffer (pH 8.0) and incubated for 15 min at 25 °C. The reaction was initiated by the addition of acetylthiocholine iodide (ATCI) or butyrylthiocholine chloride (BTCl) (25 µL). After 10 min of reaction, the absorbance at a wavelength of 405 nm was measured and the IC_50_ (μg/mL) was calculated [25].

#### 3.7.2. α-Glucosidase Inhibition Assay

Solutions were read at λ = 415 nm in a microplate reader over a one-minute interval for a total of 20 min and an acarbose standard curve was employed. The stock solution of the α-glucosidase enzyme was prepared in 2 mL at 10 U/mL of buffer for subsequent dilution. The results are expressed as (IC_50_) in μg/mL [18].

#### 3.7.3. α-Amylase Inhibition Assay

Solutions are read via spectrophotometry at λ = 515 nm using an acarbose standard curve. The α-amylase enzyme is assayed at a concentration of 0.5 mg/mL dissolved in 20 mM phosphate-buffered solution at pH 6.9 (see Supplementary Material). The results are expressed in as IC_50_ in μg/mL [18].

### 3.8. Docking Calculation Protocols

Docking simulations were performed for every compound shown in Figure 7 which belonged to the Michay (*Berberis congestiflora* Gay) ethanolic extract. Energetic minimization of each molecule was carried out using the LigPrep tool in the program Maestro Schrodinger suite v.11.8 (Schrödinger, LLC, New York, NY, USA) [39]. Crystallographic enzyme structures of Torpedo Californica acetylcholinesterase (TcAChE; PDBID: 1DX6 code [40]), human butyrylcholinesterase (hBuChE; PDBID: 4BDS code [41]), human Maltase-Glucoamylase (glucosidase; PDBID: 3TOP code [42]), and human pancreatic alpha-amylase (amylase; PDBID: 1B2Y code [43]) were obtained from the Protein Data Bank RCSB-PDB [44]. Enzyme optimizations were carried out using Maestro’s Protein Preparation Wizard. All ligands located in the catalytic pockets, as well as water molecules, were deleted prior to further processing. Appropriate ionization states for acid and basic amino acid residues, as well as polar hydrogen atoms, were considered at physiological pH = 7.4. The simulation box was set as a 26 Å cubic enclosure, and protein energy minimization was performed using the OPLS3e force field. The centroid of the selected residue was assigned according to the putative catalytic site of each enzyme and its corresponding catalytic amino acids The specific residues were as follows: Ser200 for acetylcholinesterase (TcAChE) [45,46], Ser198 for butyrylcholinesterase (hBuChE) [41,47], Asp1526 for glucosidase [42], and Asp197 for amylase [5,43]. The Glide-Induced Fit Docking protocol has been used for the final couplings [48]. Scoring of all compounds was performed with the Glide XP (extra-precision) scoring function (Schrödinger, LLC) [49] and they were filtered on the basis of the best scores and best RMS values (less than one unit as a cutting criterion) in order to obtain the potential intermolecular interactions between compounds and the enzymes, as well as the binding mode and docking descriptors. The different complexes were inspected with Visual Molecular Dynamics (VMD) and PyMOL software (PyMOL 3.1. Version 3.1.6.1) [50].

### 3.9. Statistical Analysis

Results were expressed as mean ± standard error of the mean (SEM) and were regarded as statistically significant when *p* < 0.05. Multiple comparisons were determined using ANOVA in Prism (version 6.0, (GraphPad Software Inc., San Diego, CA, USA).

## 4. Conclusions

In this study, the phenolic and anthocyanin composition of *B. congestiflora* extracts was characterized, along with their potential as antioxidants and as therapeutic agents against chronic non-communicable diseases. Overall, the results broaden our understanding of secondary metabolites in these native Berberis species and support their bioactivity, particularly their biphasic vascular effects (contraction/vasodilation) and their possible use as supplements for Alzheimer’s disease (through AChE inhibition) and for diabetes or metabolic syndrome (via glucosidase and amylase inhibition), thereby providing a foundation for further research. Future work should prioritize assessing the biological activities of key, yet-unidentified, pure anthocyanin compounds in animal and cellular models, as well as performing pharmacodynamic and pharmacokinetic studies to clarify their mechanisms of action. In addition, developing biotechnological or chemical synthesis approaches based on the identified compounds may help to improve their potential for future biological and therapeutic applications.

## Figures and Tables

**Table 1:** Radical scavenging activity of 1,1-diphenyl-2-picrylhydrazyl radical (DPPH), ABTS radicals, total phenolic content (TPC), total anthocyanin content (TAC), total flavonoid content (TFC), cholinesterase inhibition capacity, and glucosidase and amylase inhibition capacity of the hydroalcoholic extract of *B. congestiflora* (BE).

Sample	DPPH ^a^	ABTS ^a^	ORAC ^b^	FRAP ^b^	TPC ^c^	TAC ^d^	TFC ^e^	AChE ^f^	BuChE ^f^	^f^ α-Glucosidase	^f^ α-Amylase
BE extract	5.32 ± 0.5	6.78 ± 0.04	175.9 ± 3.43	148.7 ± 0.03	76.35 ± 0.01	32.26 ± 1.23	63.2 ± 0.2	7.33 ± 0.32	19.45 ± 0.32	243.23 ± 0.3	27.21 ± 0.03
Gallic acid	2.30 ± 0.5	16.5± 0.04	-	-	-	-	-	-	-	-	-
Acarbose	-	-	-	-	-	-	-	-	-	138.9 ± 0.01	10.04 ± 0.02
Galantamine	-	-	-	-	-	-	-	0.402 ± 0.02 ^e^	5.45 ± 0.01	-	-
Quercetin	12.25 ± 0.6	15.65 ± 0.05	-	-	-	-	-	-	-	-	-

^a^ DPPH antiradical and ABTS activities are expressed as IC_50_ in μg/mL. ^b^ Expressed as μmol Trolox/g dry fruit. ^c^ Total phenolic content (TPC) expressed as mg gallic acid equivalent (GAE/g dry weight).^d^ Total anthocyanin content (TAC) expressed as mg c3g equivalent/g dry weight. ^e^ Total flavonoid content (TFC) expressed as mg quercetin/g dry weight. ^f^ Inhibitory enzymes of cholinesterases, α-glucosidase, and α-amylase enzymes in IC_50_ expressed in µg/mL. Values in the same column are significantly different (*p* < 0.05). Dash (-) means not tested (i.e., not applicable).

**Table 2:** Identification of phenolic compounds in *Michay* fruits by LC-DAD, LC–MS, and MS/MS data.

Peak	Rt (min)	HPLC DAD λ Max (nm)	ESI Mode	[M-H]^–^ (*m*/*z*)	MS-MS Ions (*m*/*z*)	Tentative Identification
1	2.2	268, 357 sh, 503	+	611	287	Cyanidin 3,5-O-diglucoside, (Cyanidin-3,5-O-di-β-D-glucopyranoside)
2	3.1	268, 357 sh, 503	+	595	287	Cyanidin 3-O-[6″-O-(p-coumaroyl)] glucoside (Cyanidin-3-O-[6″-O-(p-coumaroyl)] glucopyranoside)
3	3.3	275, 343 sh, 512	+	641	317, 302	Petunidin 3,5-O-diglucoside, (Petunidin-3,5-O-di-β-D-glucopyranoside)
4	6.1	275, 343 sh, 512	+	479	317, 302	Petunidin 3-O-glucoside * (Petunidin-3-O-β-D-glucopyranoside)
5	8.2	268, 357 sh, 503	+	609	463, 301, 286	Peonidin 3-O-rutinoside
6	9.8	275, 341 sh, 512	+	465	303, 257	Delphinidin 3-O-glucoside * (Delphinidin-3-O-β-D-glucopyranoside)
7	11	268, 357 sh, 503	+	463	301, 286	Peonidin 3-O-glucoside * (Peonidin-3-O-β-D-glucopyranoside)
8	11.2	268, 357 sh, 503	+	505	317, 302	Peonidin 3-O-[6″-O-(acetyl)]-glucoside, (Peonidin-3-O-([6″-O-(acetyl)]-β-D-glucopyranoside)
9	12.1	246, 310	−	515	353, 191, 179	Di-caffeoyl-quinic acid (4,5-O-di-caffeoylquinic acid)
10	13.2	246, 310	−	353	191, 179	3-O-Caffeoylquinic acid, (Chlorogenic acid) *
11	12.3	268, 357 sh, 503	+	625	317, 302	Peonidin 3,5 O-di-glucoside, (Peonidin-3,5-O-di-β-D-glucopyranoside)
12	14.6	275, 343 sh, 512	+	639	331, 299, 179	Malvidin 3-O-rutinoside
13	16	278, 503	+	449	287, 213, 147	Cyanidin-3-O-glucoside *, (Cyanidin-3-O-β-D-glucopyranoside)
14	16.3	275, 343 sh, 512	+	493	331, 299, 179	Malvidin 3-O-glucoside *, (Malvidin-3-O-β-D-glucopyranoside)
15	16.7	275, 343 sh, 512	+	331	299, 179	Malvidin
16	17.2	255, 354	−	463	301, 179, 151	Quercetin-3-O-galactoside * (Quercetin-3-O-β-D-galactopyranoside, Hyperoside)
17	18.7	255, 354	−	505	463, 301, 179, 151	Quercetin-3-O-([6″-O-(acetyl)]-glucoside), Quercetin-3-O-([6″-O-(acetyl)]-β-D-glucopyranoside)
18	22.5	265, 354	+ −	477, 479	955 (2M-H) 315, 300	Isorhamnetin-3-O-glucoside (Isorhamnetin 3-O-β-D-glucopyranoside *
19	23.4	255, 354	−	623	315	Isorhamnetin 3-O-rutinoside (Narcissin)
20	23.8	255, 354	−	463	301, 179, 151	Quercetin-3-O-glucoside * (quercetin 3-O-β-D-glucopyranoside
21	24.2	254, 354	−	609	301, 179, 151	Quercetin 3-O rutinoside, (Rutin) *
22	24.9	255, 354	−	447	287	Luteolin 7-O-glucoside (Luteolin 7-O-β-D-glucopyranoside
23	25.3	255, 354	−	597	287	Phloretin 3′,5′-Di-C-glucoside
24	25.7	255, 354	−	519	477, 315, 179, 151	Isorhamnetin-3-O-([6″-O-(acetyl)]-glucoside),(Isorhamnetin-3-O-([6″-O-(acetyl)]-β-D-glucopyranoside)
25	26.1	255, 354	−	529	367	4-Caffeoyl-5-feruloylquinic acid *
26	26.5	240–290	−	373	171	8-Hydroxypinoresinol
27	28.3	246, 310	−	371	209, 742 (2M-H^−^)	5-Hydroxyferulic acid
28	29.7	265, 354	+ −	315, 317	300, 179, 151	Isorhamnetin *
29	30.2	265, 354	+ −	315, 317	300, 179, 151	Rhamnetin *
30	31.3	246, 310	−	339	295	Unknown
31	31.6	255, 354	+ −	301, 303	295	Quercetin *

Compounds marked by * were identified by co-spiking experiments using UHPLC-DAD (Figure 4).

**Table 3:** Binding energies obtained from docking experiments of selected compounds from the hydroalcoholic extract of *Berberis congestiflora* Gay (BE), as well as the known inhibitors galantamine and acarbose over acetylcholinesterase (*Tc*AChE), butyrylcholinesterase (*h*BChE), glucosidase, and α-amylase.

Compound	Binding Energy (kcal/mol) Acetylcholinesterase	Binding Energy (kcal/mol) Butyrylcholinesterase	Binding Energy (kcal/mol) Glucosidase	Binding Energy (kcal/mol) α-Amylase
Peonidin 3-O-glucoside	−14.837	−12.461	−15.462	−8.597
Peonidin 3-O-rutinoside	−15.024	−13.129	−15.697	−11.606
Malvidin 3-O-glucoside	−15.793	−12.944	−16.176	−9.793
Malvidin 3-O-rutinoside	−11.632	−13.472	−12.436	−14.120
5-Hydroxyferulic acid	−8.647	−6.753	−8.017	−5.147
4-Caffeoyl-5-feruloylquinic acid	−10.068	−10.973	−10.555	−7.167
Quercetin-3-O-glucoside	−13.287	−12.808	−13.390	−11.191
Isorhamnetin-3-O-rutinoside	−15.658	−13.922	−11.544	−10.766
Galantamine	−12.989	−7.125	---------	---------
Acarbose	---------	---------	−18.591	−12.626

## Data Availability

The original and pure contributions shown in this study are included in the article/Supplementary Materials. Further inquiries can be directed to the main authors.

## Supplementary Materials

The following supporting information can be downloaded at https://www.mdpi.com/article/10.3390/plants15030352/s1. Section S1: Full MS and MS-MS spectra of some selected compounds identified in Michay berries. Section S2: In vitro Bioassays Protocols.
