# Supplementary material for: Polyphenolic Profile and Antioxidant and Aortic Endothelium Effect of Michay (Berberis congestiflora Gay) Collected in the Araucanía Region of Chile
Source: Plants (Basel). 2026 Jan 23;15(3):352. doi: 10.3390/plants15030352 (PMC12899593; doi:10.3390/plants15030352)
Supplement: Supplementary file 1 [file plants-15-00352-s001.zip › plants-4072402-supplementary.pdf]

Supplementary material

Section S1. Full MS and MS-MS Spectra of Some Selected Compounds Identified in *Michay* Berries.

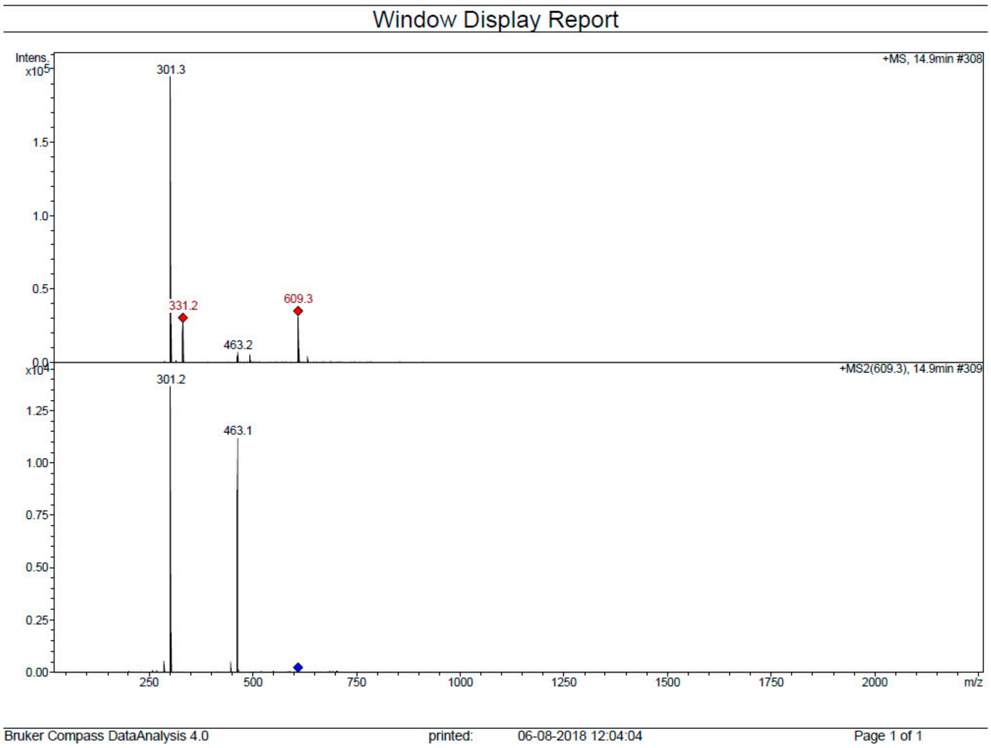

Window Display Report

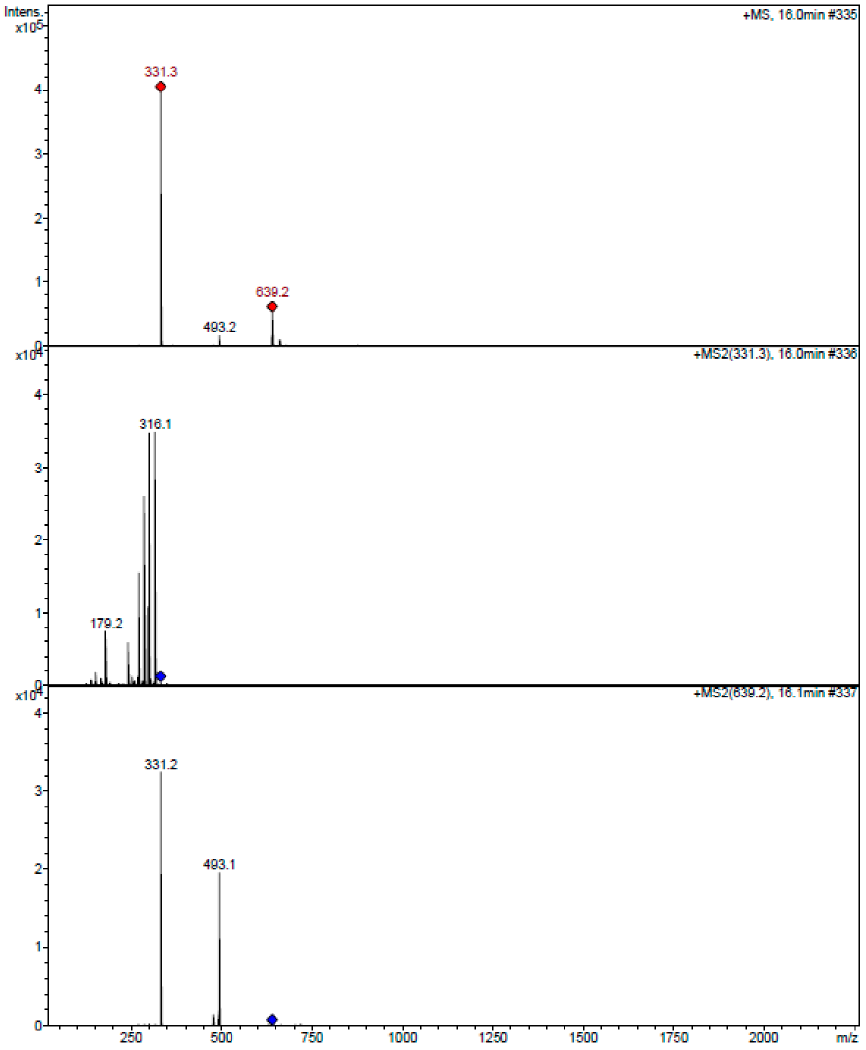

Window Display Report

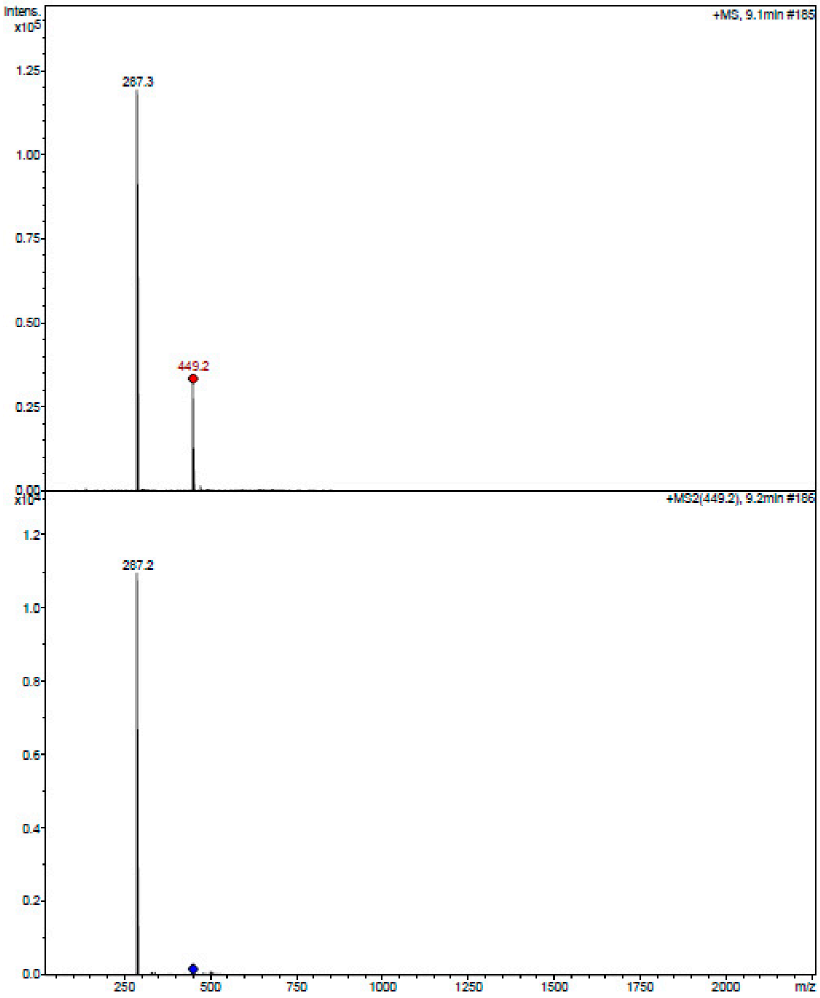

Window Display Report

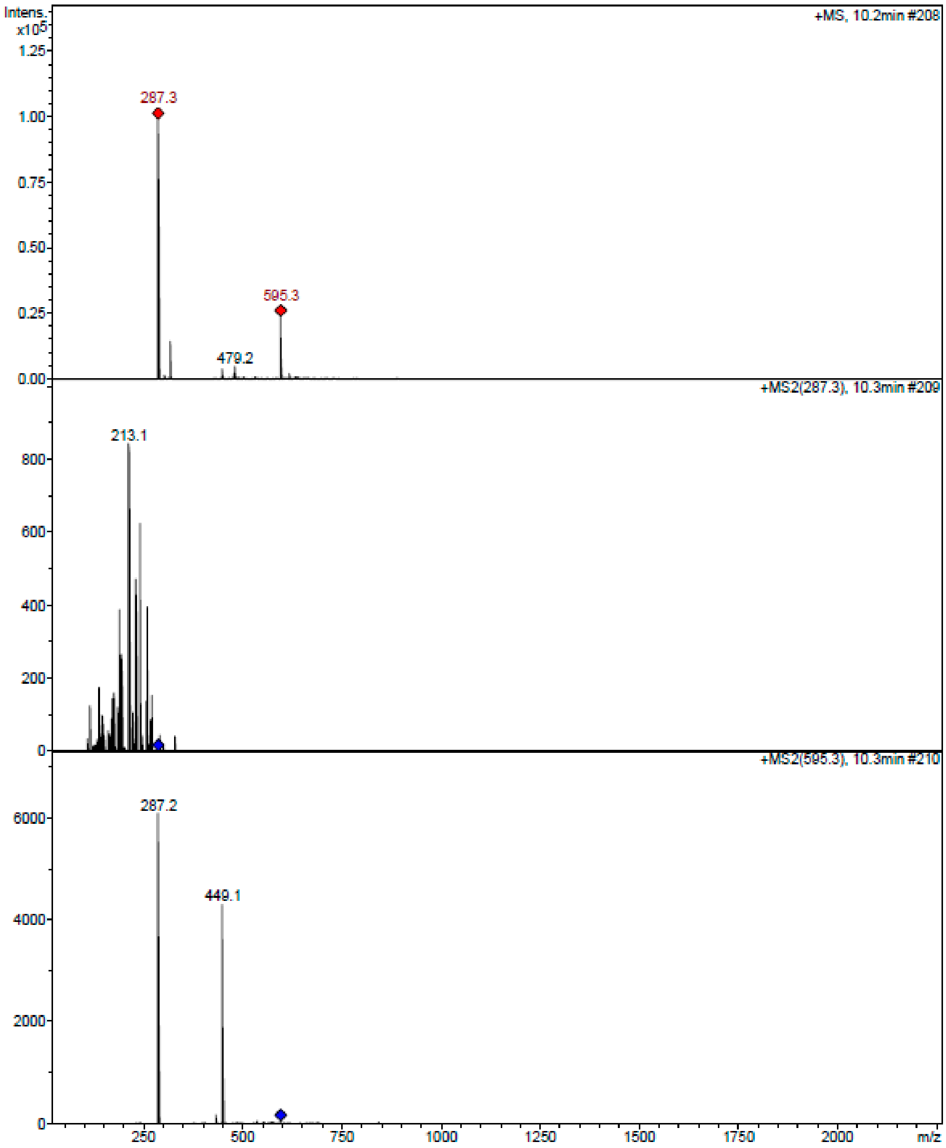

Window Display Report

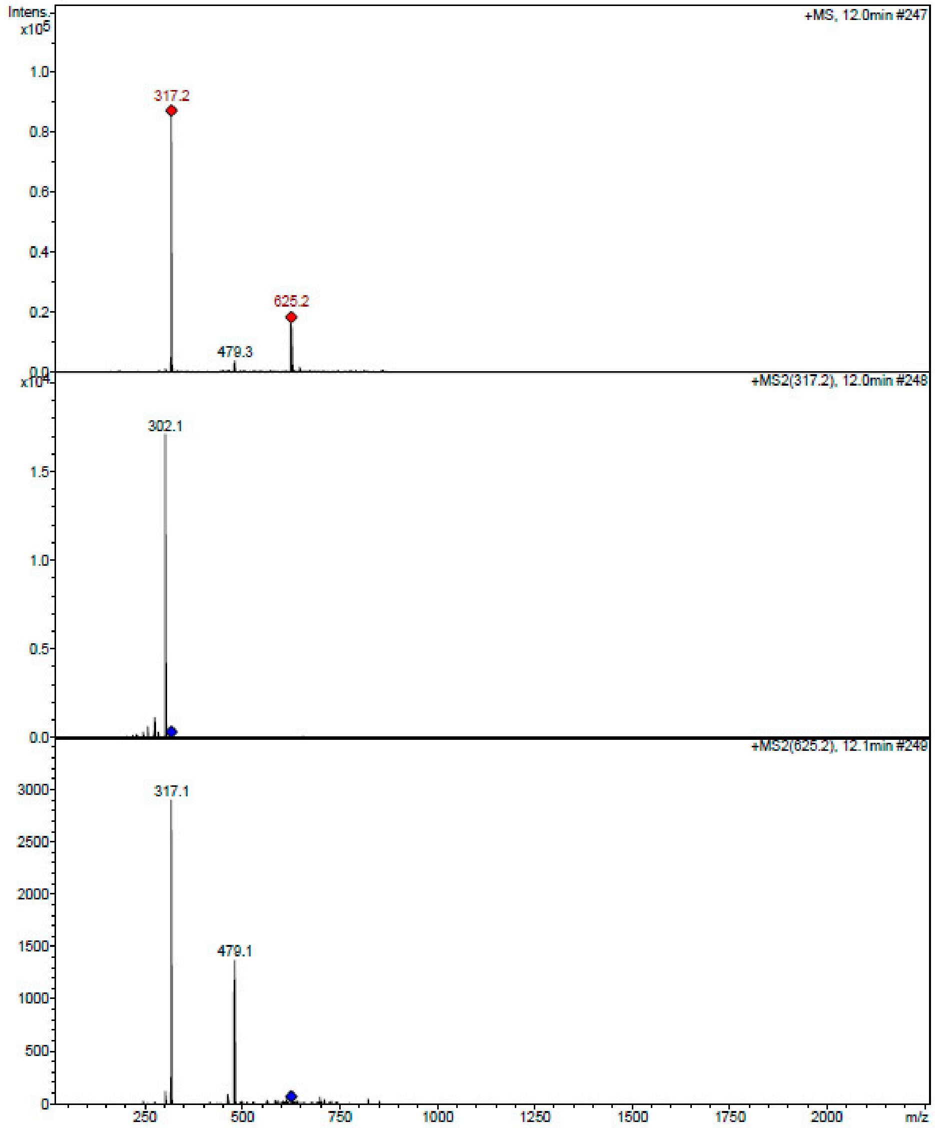

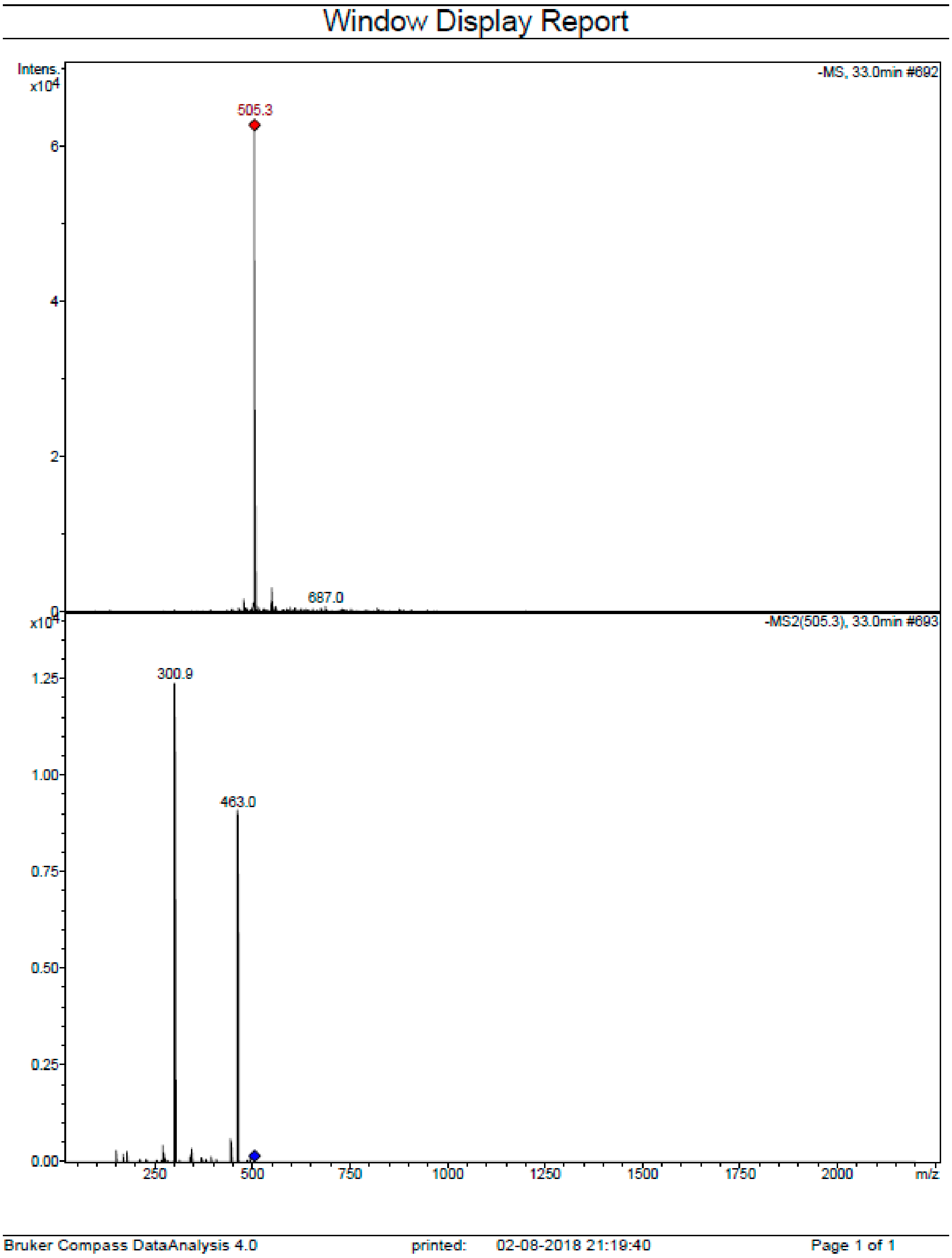

Section S2. In vitro Bioassays Protocols

Determination of ORAC

75 mM PBS Buffer pH 7.4 For one liter of buffer, weigh 3.97 g of NaH_2PO_4 and 6.532 g of Na_2HPO_4 and make up to 1000 mL with deionized H_2O. 18 mM AAPH Prepare 5 minutes before use.
For 5 mL of PBS buffer, add 25 mg of AAPH. 108 nM Fluorescein Prepare a 1 mM fluorescein stock solution. To do this, weigh 1.88 mg in 5 mL of PBS buffer. Then take 2.16 µL of the stock solution and make up to 20 mLwith PBS buffer for a final concentration of 108 nM.

Microplate reaction:

Add-45 µL of sample or standard, Add-175 µL of 108 nM fluorescein, Incubate at 37°C for 30 min.

Add-50 µL of 18 mM AAPH, Read for 2 h every 2 min at an excitation wavelength of 480 nm and an emission wavelength of 520 nm.

The curve ranges from 1-20 µM in microtubes.

Controls:

Blank: (No fluorescein)
-Control: No inhibitor
-Control: Trolox

Determination of Ferric Reduction Complex (FRAP)

Reagents:
-12 mM sodium acetate trihydrate buffer, pH 3.6: Mix 1.64 g of CH_3COONa with 16 mL of glacial CH3COOH for 1 liter of solution (make up to volume with distilled water).

-10 mM TPTZ solution (2,4,6-tripyridyl-s-triazine): Weigh 0.031 g into a 10 mL flask and make up to the mark with diluted HCl (40 mM). It is important to prepare this solution on the same day it will be used.
-20 mM $FeCl_3 \cdot 6H_2O$ solution: Weigh 0.054 g of $FeCl_3 \cdot 6H_2O$ into a 10 mL flask and make up to the mark with distilled water.

-FRAP reagent: Mix 1020 µL of pH 3.6 buffer, 100 µL of 10 mM TPTZ, and 100 µL of 20 mM $FeCl_3.6H_2O$.

Microplate reaction:

Add 10 µL of sample (Standard), Add 290 µL of FRAP reagent, wait 60 min, then read at 593 nm.

Determination of Antioxidant Capacity Using DPPH Radical Bleaching

Reagents:

Prepare 156 µM DPPH stock solution (methanol/ethanol) Prepare
the sample in methanol/ethanol (As many dilutions as needed)
Prepare a gallic acid curve from 20-200 µM in microcentrifuge tubes

Microplate Reaction
Add-50 µL (standard or sample), Add-150 µL of DPPH solution and Incubate for 30 min in the dark, then measure the absorbance at 517 nm

Determination of Antioxidant Activity Using ABTS Radical Bleaching

Reagents:
Prepare ABTS stock solution by mixing ABTS and sodium persulfate in distilled H_2O,
considering a final concentration of 7 mM ABTS and a final concentration of 3.6 mM sodium persulfate ($Na_2S_2O_8$). Let the reaction incubate at room temperature and in the dark for 24 h.
Then, the following day, dilute the ABTS stock solution to a final concentration of 169 µM. Prepare the sample in methanol/ethanol (Dilutions as needed)
Prepare a gallic acid curve and quercetin curve from 20-200 µM in microcentrifuge tubes

Microplate Reaction
Add-50 µl (standard or sample), Add-150 µL of ABTS
Incubate for 30 min in the dark, Then, measure the absorbance at 732 nm

α-Amylase Inhibition Protocol

Reagents:
Phosphate buffer (20 mM pH 6.9)
For 250 ml deionized water
-456.1 mg $NaH_2PO_4*H_2O$
-240 mg Na_2HPO_4
-87.7 mg NaCl
DNS 96 mM NaK 40%

First, a 0.5 N NaOH solution is weighed.
Then, 40 g of NaK is added little by little to 80 mL of this NaOH, followed by 2.19 g of DNS and then diluted to 100 mL (dissolved with constant stirring and temperature) until the solution is completely homogeneous.
Starch substrate 0.5%
-250 mg of starch soluble in deionized water
-Dissolve carefully in a water bath, Add-0.5 mg/mL α-amylase enzyme, Add-2.5 mg in 5 mL of buffer
Reaction (perform in triplicate):
Add 100 µL of sample, Add 100 µL of enzyme, incubate for 10 min at 25°C, Add 100 µL of starch
Incubate for 10 min at 25°C, Add 200 µL of DNS, Place samples in a water bath (90°C) for 5 min
Cool to room temperature, then read at 540 nm

α-Glucosidase inhibition Protocol

Reagents:

100 mM phosphate buffer pH 6.9, In 200 mL of deionized H_2O
Add -2.30 g NaH_2PO_4
Add-1.19 g Na_2HPO_4
0.5 U/ml α-glucosidase
Make a stock solution of 10 U/mL(1 mg/mL)
-Add 250 µL and dilute to 5 ml of buffer
Add 5 mM P-nitrophenyl-α-D-glucopyranoside
-Weigh 0.0075 g in 5 mL of deionized H_2O
200 mM Sodium Carbonate (Na_2CO_3)
-Mass 1.06 g of Na_2CO_3 in 50 mL of deionized H_2O
Reaction in test tubes:
Add-600 µL of 100 mM phosphate buffer pH 6.9, add -250 µL of p-NPG, add -100 µL of sample

Preincubate 5 min at 37°C, add -50 µL of 0.5 U/mL α-glucosidase enzyme

Incubate 15 min at 37°C, add -1000 µL of Na_2CO_3, Read at 400 nm

AChE and BuChE Enzyme Inhibition Protocols

Reagents: 50 mM Tris-HCl buffer, pH 8.0, For 500 mL
Add-Trizma base 3.0238 g, add -37% HCl 1.1 mL

3 mM DTNB
For 100 mL (in buffer)
Add-0.1138 g of DTNB
Add-0.585 g of NaCl
Add-0.466 g of $MgCl_2$

5 mM AChE substrate
Add-7.23 mg in 5 mL of deionized water
Add 0.3 U/mL AChE enzyme
For 5 ml of buffer

Add -3 uL of stock (500 U/mL) *

*0.28 U/mL BuChE enzyme
For 5 mL of buffer
Add-13 uL of stock (100 U/mL)
Add 5 mM BuChE substrate
Add -7.93 mg in 5 mL of deionized water *

*NOTE
Blank (add everything except the enzyme) Control
(-) (Add everything except the sample)
Reaction:

On a kinetic plate, Add-25 uL sample, add -125 uL LDTNB, add -25 uL Enzyme solution
Incubate for 15 min at 37°C, add -25 uL substrate, Read for 20 min every 1 min at 412 nm and 37°C

Determination of Total Phenolic Content

Reagent Preparation
- 10% (v/v) F-C Reagent: Mix 1 mL of RF-C in 10 mL of distilled water.
- 5% (w/v) Na_2CO_3: Mix 5 g of Na_2CO_3 in a 100 mL flask and make up to the mark with distilled water.

Microplate reaction:
- 10 µL sample (standard)
- 150 µL H_2O
- 12.5 µL Folin's reagent

Incubate for 5 min at 37°C, add - 37.5 µL 5% $NaCO_3$, Incubate for 30 min at 37°C, then Read at 765 nm

Curve: Gallic acid (GA) as standard (20–160 µg/mL methanolic solutions)
